# Adaptation induced by self-targeting in a type I-B CRISPR-Cas system

**DOI:** 10.1074/jbc.RA120.014030

**Published:** 2020-07-28

**Authors:** Aris-Edda Stachler, Julia Wörtz, Omer S. Alkhnbashi, Israela Turgeman-Grott, Rachel Smith, Thorsten Allers, Rolf Backofen, Uri Gophna, Anita Marchfelder

**Affiliations:** 1 Biology II, Ulm University, Ulm, Germany; 2 Bioinformatics Group, Department of Computer Science, University of Freiburg, Freiburg, Germany; 3 Department of Molecular Microbiology and Biotechnology, George S. Wise Faculty of Life Sciences, Tel Aviv University, Tel Aviv, Israel; 4 School of Life Sciences, University of Nottingham, Nottingham, UK; 5 Signalling Research Centres BIOSS and CIBSS, University of Freiburg, Freiburg, Germany

**Keywords:** CRISPR-Cas, type I-B, archaea, Haloferax volcanii, adaptation, self-targeting, crRNA, homologous recombination, naive adaptation

## Abstract

*Haloferax volcanii* is, to our knowledge, the only prokaryote known to tolerate CRISPR-Cas–mediated damage to its genome in the WT background; the resulting cleavage of the genome is repaired by homologous recombination restoring the WT version. In mutant *Haloferax* strains with enhanced self-targeting, cell fitness decreases and microhomology-mediated end joining becomes active, generating deletions in the targeted gene. Here we use self-targeting to investigate adaptation in *H. volcanii* CRISPR-Cas type I-B. We show that self-targeting and genome breakage events that are induced by self-targeting, such as those catalyzed by active transposases, can generate DNA fragments that are used by the CRISPR-Cas adaptation machinery for integration into the CRISPR loci. Low cellular concentrations of self-targeting crRNAs resulted in acquisition of large numbers of spacers originating from the entire genomic DNA. In contrast, high concentrations of self-targeting crRNAs resulted in lower acquisition that was mostly centered on the targeting site. Furthermore, we observed naïve spacer acquisition at a low level in WT *Haloferax* cells and with higher efficiency upon overexpression of the Cas proteins Cas1, Cas2, and Cas4. Taken together, these findings indicate that naïve adaptation is a regulated process in *H. volcanii* that operates at low basal levels and is induced by DNA breaks.

Bacteria and Archaea have developed CRISPR-Cas systems as a means to fend off invading genetic elements in a sequence-specific manner (1, 2). This specificity is provided by the spacer, a portion of the foreign nucleic acid taken up into a CRISPR locus during adaptation (1, 3). Upon transcription of the CRISPR array and subsequent processing of the transcript, the resulting mature CRISPR RNAs (crRNAs), together with one or multiple Cas proteins, form an effector complex capable of orchestrating a defense reaction termed interference (1, 4) and degrade the invader´s nucleic acid.

The mechanisms of processing and interference exhibit significant variability within the increasing variety of CRISPR-Cas systems described today. Currently, CRISPR-Cas systems are divided in two classes, six types and over 30 subtypes, each exhibiting variabilities in their CRISPR-Cas immune response mechanisms (5). The adaptation process, however, seems to be more conserved, as it requires the proteins Cas1 and Cas2 in all systems studied to date (6–11). (The term adaptation is used in the CRISPR-Cas context for acquisition of new spacers and their integration into the CRISPR locus.) Two types of adaptation have been observed so far: Naïve adaptation, which is triggered upon first contact with a foreign nucleic acid and primed adaptation. (The term naïve adaptation is used in the CRISPR-Cas context for the acquisition of spacers without the interference reaction and without a crRNA that matches the prespacer sequence fully or partially.) The latter occurs during infection with an invader against which the cell is already immune, and in type I systems requires involvement of the multiprotein effector complex Cascade (CRISPR-associated complex for antiviral defense) and Cas3 (12, 13). A number of studies have elucidated this process in recent years, yielding a mechanistic model in which degradation products generated by the nuclease Cas3 can be utilized as prespacers by the adaptation machinery. This alliance of defense and acquisition stage results in a positive feedback loop: New spacers boost the defense reaction against the present invader, resulting in even more cleavage products suitable for further acquisition events, while promoting interference (14–18). Accidental acquisition of spacers from the cellular genome during adaptation has been reported by several studies with different CRISPR-Cas systems and different organisms showing that this is not a rare phenomenon (17, 19–21). However, the resulting interference reaction against chromosomal DNA is usually highly toxic (22–25).

We have shown previously that self-targeting has no detrimental effects in an *H. volcanii* WT strain (26). The influence on cell fitness and the target gene depended on the crRNA concentration. In a *Haloferax* mutant strain depleted of endogenous crRNAs, expression of a crRNA from a high-copy plasmid led to a very efficient self-targeting reaction; the transformation efficiency with the crRNA-encoding plasmid was reduced, and 99% of growing colonies exhibited deletions in the target gene, thereby abolishing further self-targeting. In contrast, expression of the crRNA from a low-copy plasmid only slightly reduced transformation efficiency, and only a low number of cells had the targeting site deleted (26). Here, we utilize self-targeting to gain deeper insights into the adaptation mechanism in *H. volcanii*. The CRISPR-Cas system of *H. volcanii* has been studied in great detail (27). It is classified as subtype I-B and comprises three CRISPR loci and one *cas* gene cassette (Fig. 1). Although mature crRNAs from all loci as well as the Cascade proteins Cas5–8b are constitutively expressed (28–30), proteins from the adaptation machinery are not detectable under standard growth conditions (30). This indicates that in contrast to expression and interference, the adaptation process is regulated and needs to be induced.

**Figure 1.**
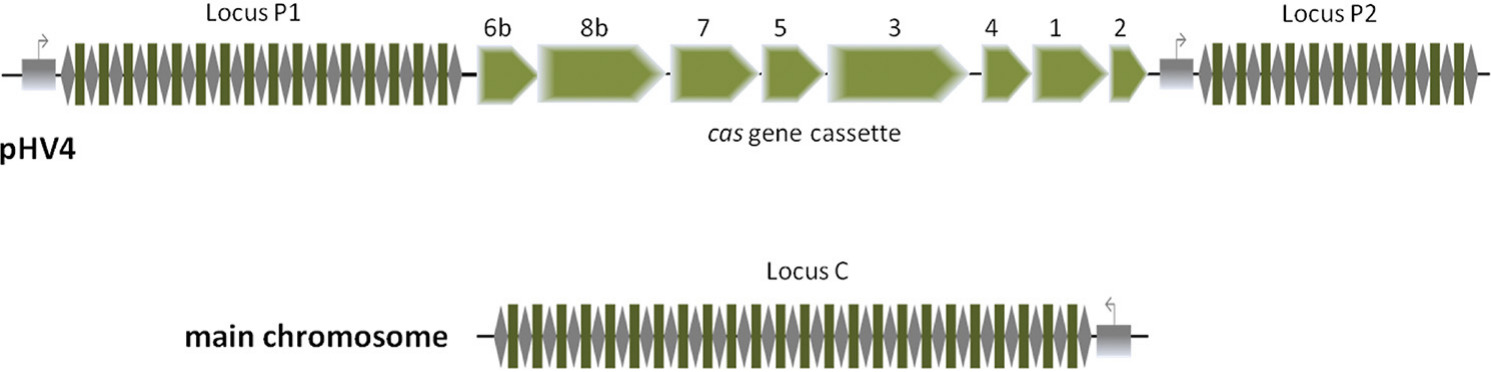
**CRISPR-*cas* genes encoded in *Haloferax volcanii*.** *H. volcanii* encodes a CRISPR-Cas type I-B system. The *Haloferax* genome consists of one main chromosome and three chromosomal plasmids (35). The *cas* gene cluster encoding the Cas1-Cas8b proteins is flanked by the two CRISPR loci P1 and P2 on the chromosomal plasmid pHV4. The third CRISPR locus C is encoded on the main chromosome.

Here, we observe that targeting a chromosomal gene induces acquisition of spacers from all genomic elements. Employing high-throughput sequencing (HTS), we identify clear hotspots for spacer acquisition located near transposase genes and, to a lesser extent, at highly transcribed regions, indicating a bias for sites with a higher occurrence of free DNA ends. Furthermore, we report naïve adaptation; both WT and Δ*cas6b Haloferax* strains acquire spacers at similar levels also without self-targeting, confirming that the process is truly naïve adaptation and does not require interference activity. Overexpression of the adaptation machinery increased naïve adaptation, which was independent of the cognate PAM sequence.

## Results

### Self-targeting triggers adaptation in H. volcanii

**Figure 2.**
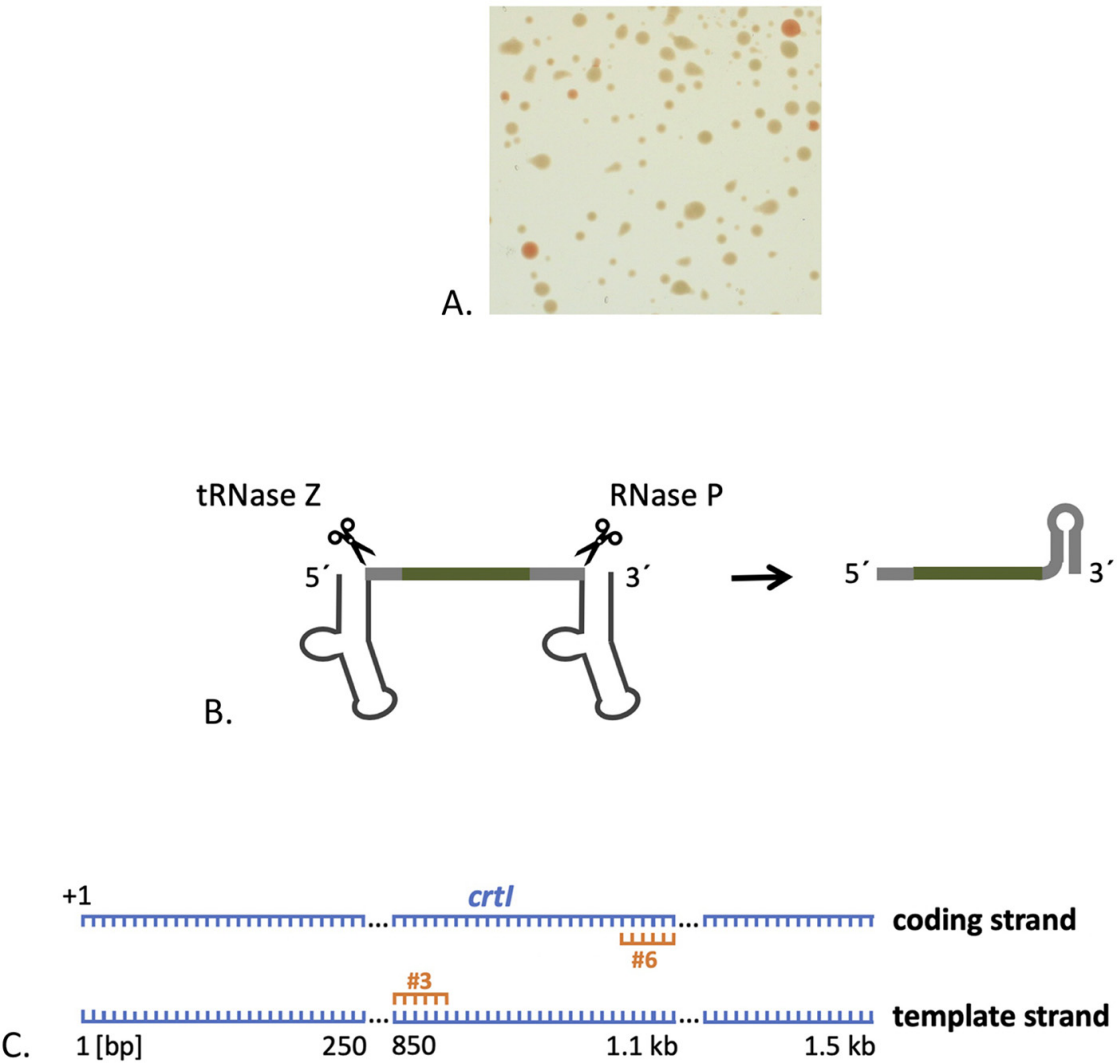
**Targeting the chromosomal gene *crtI*.** *A*, colonies without an active phytoene dehydrogenase are *white*. Colonies after transformation with a high-copy plasmid encoding crtI#3. Although some colonies exhibit the natural *red color*, the majority of colonies are completely devoid of pigmentation. *B*, production of crRNAs independent of Cas6b. The genes for the mature crRNA (containing a spacer, an 8-nt-long 5′ handle and a 22-nt-long 3′ handle) are flanked by genes for t-elements; they are transcribed together into a precursor RNA. The t-elements within the transcript fold into tRNA-like structures that are recognized and processed by the cellular tRNase Z and RNase P proteins, generating a mature crRNA. *C*, location of crRNA binding sites in *crtI*. The gene *crtI* is shown with both strands, starting with the first nucleotide of the ORF (+1); crRNAs crtI#3 and crtI#6 are shown in *orange*.

To analyze adaptation under self-targeting conditions, we designed a crRNA to target the nonessential *crtI* gene that is located on the main chromosome and codes for phytoene dehydrogenase. Cells with a defect in this gene have a white colony phenotype instead of the natural red color (Fig. 2*A*), thus mutants are easily visible. Because previous experiments have shown that self-targeting in a WT background is not efficient, a *cas6b* gene deletion strain (Δ*cas6b*) was used. In this strain, endogenous crRNAs cannot be generated because the gene for the crRNA processing enzyme Cas6b is deleted.

**Figure 3.**
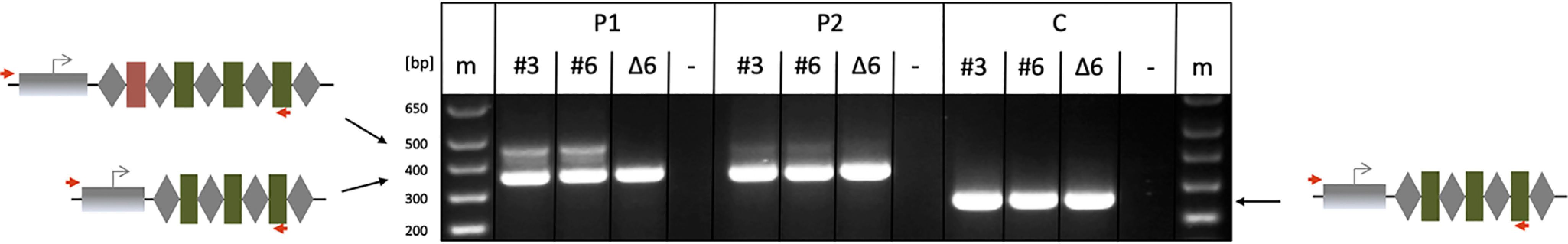
**Integration of new spacers into CRISPR loci.** Whereas new spacers are integrated in locus P1 and P2 (*lanes P1* and *P2*), no spacers have been inserted in locus C (*lane C*). PCR was performed to amplify the region between leader and spacer2 (for locus P1 and locus P2) or spacer1 (for locus C) on genomic DNA from Δ*cas6b* strains transformed with plasmid expressing either crRNA crtl#3 or crtI#6 from a low-copy plasmid (pTA352) (*lanes crtl#3* and *crtI#6*, respectively). *Lanes* Δ*6*: genomic DNA from strain Δ*cas6b* was used that was not transformed with any plasmids; *lanes* −: control reaction without addition of template DNA; *lane m*: DNA size marker, sizes are given in bp at the left. The 5´ ends of the CRISPR loci are shown schematically at the sides. Primer binding is indicated by *arrows*; the newly added spacer is shown in *orange*.

To express the targeting crRNA in the Δ*cas6b* strain, the gene encoding the mature crRNA form was cloned between two tRNA-like elements (t-elements) (Fig. S1). The t-element–crRNA transcript is processed by the endogenous tRNA processing enzymes RNase P and tRNase Z yielding the mature, functional crRNA (Fig. 2*B*) (31, 32). The Δ*cas6b* strain was transformed with 1) a plasmid encoding a crRNA (crtI#6) targeting the coding strand or 2) a plasmid targeting the template strand (crtI#3) of the *crtI* gene (Fig. 2*C*). Both crRNAs were expressed from a low-copy plasmid (pTA352), resulting in low cellular crRNA concentrations (26). To monitor integration of new spacers into one of the three CRISPR RNA loci, PCR was performed on genomic DNA isolated from the transformed cells using primers that bind to the leader sequence and to one of the spacers of each locus (Fig. S2). For loci P1 and P2, a larger product could be obtained corresponding in size to one additional repeat-spacer unit (Fig. 3). No such product could be observed for the CRISPR gene on the main chromosome, locus C, indicating that no new spacers were acquired (Fig. 3). Thus, self-targeting results in adaptation in the two CRISPR loci flanking the *cas* gene cluster, but not in the locus on the main chromosome.

### Spacers are derived throughout the entire chromosome

**Figure 4.**
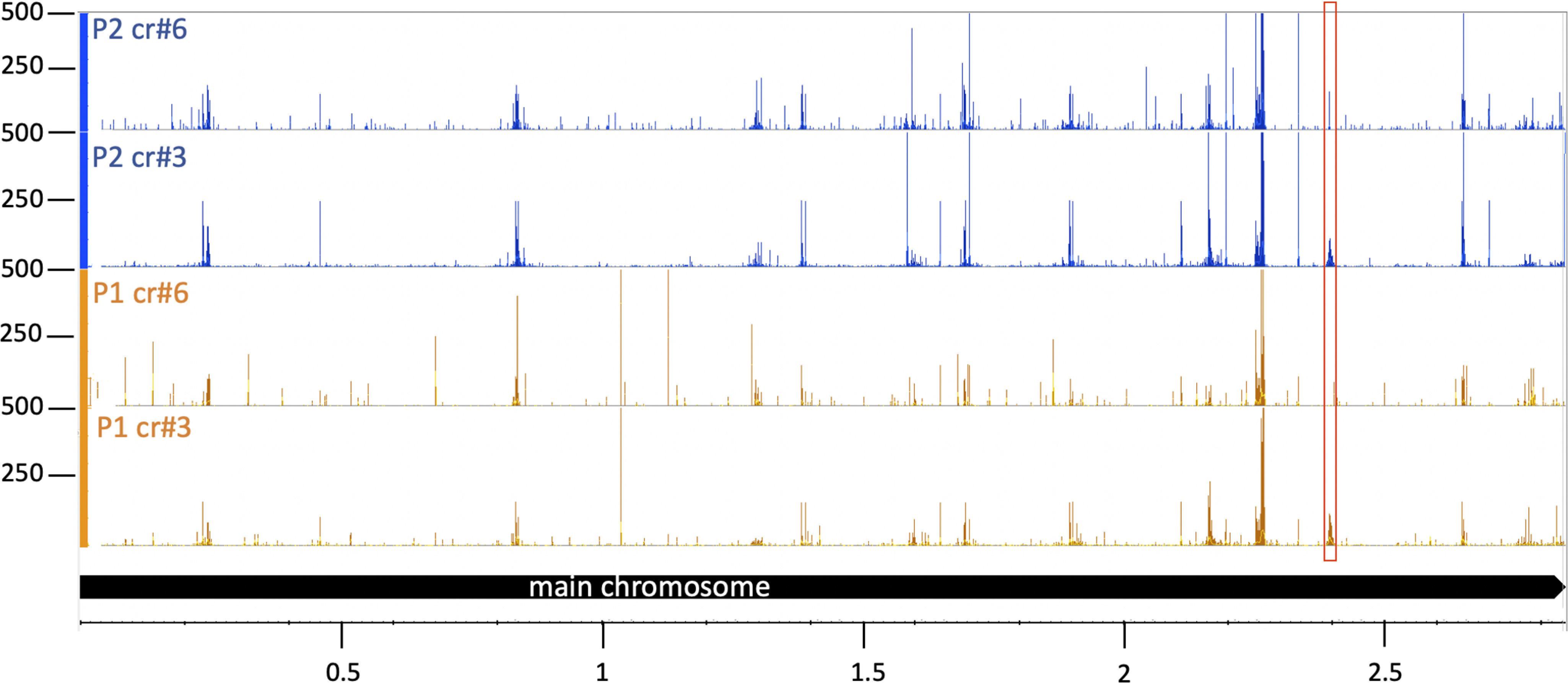
**New spacers originate from throughout the main chromosome.** HTS analysis revealed that spacers originated from regions throughout the main chromosome. New spacers integrated into CRISPR loci P1 and P2 were sequenced and mapped to the main chromosome. Chromosome coordinates are shown at the bottom in Mb; panels P1 (*orange*): new spacers integrated in locus P1; panel P2 (*blue*): new spacers integrated in locus P2; panels cr#3: targeting with crRNA crtI#3; panels cr#6: targeting with crRNA crtI#6; number of spacer reads are shown at the *left*. The crRNA targeting site is indicated with a *red box*.

**Figure 5.**
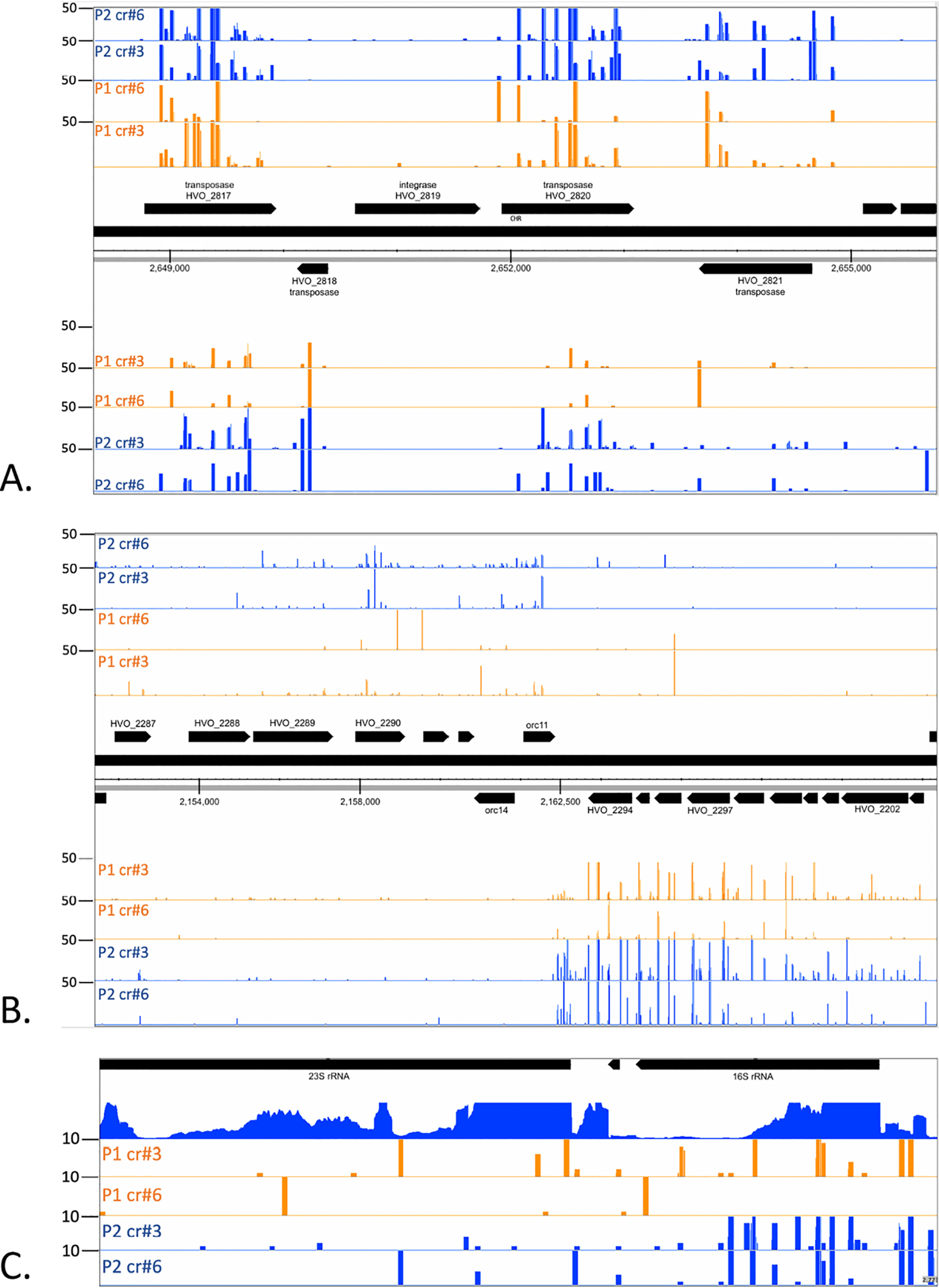
**Hotspots for spacer acquisition.** *A*–*C*, major hotspots for spacer acquisition were detected at genes for transposases (*panel A*), *orc11* and *orc14* genes (*panel B*) and to a lesser extent, highly transcribed regions like rRNA genes (*panel C*). *A*, many spacers originate from transposase genes and both strands are sources for new spacers. *B*, spacers also originate from regions flanking the genes *orc11* and *orc14*. *C*, rRNA genes are highly transcribed (see reads from panel RNA) and constitute also a source for new spacers. *Panels P1*: spacers integrated in locus P1 upon targeting with crRNA crtl#3 (cr#3) or crtl#6 (cr#6); *panels P2*: spacers integrated in locus P2 upon targeting with crRNA (cr#3) or crtl#6 (cr#6); RNA: transcriptome data. Strand-specific spacer acquisition is shown in all panels. Number of spacer reads are shown at the *left*.

To determine the origin of the newly acquired spacers integrated into locus P1 and P2, spacers were amplified using PCR and HTS was carried out. Mapping of the spacer sequences to the genome showed that they were derived from throughout the entire chromosome (Fig. 4 for the main chromosome and Fig. S3 for the small chromosomes), with similar hotspots of spacer acquisition in all samples. The majority of these hotspots were located at or near genes encoding transposases (Fig. 5*A* and Table S1), one hotspot was found near the *orc11* and *orc14* genes (Fig. 5*B*) and a smaller hotspot was located at the highly transcribed rRNA genes (Fig. 5*C*). Newly acquired spacers in cells with crtI#3 targeting and crtI#6 targeting showed highly similar patterns except for the targeting site. The consensus PAM for spacers acquired from the main chromosome and from all three chromosomal plasmids (pHV1, pHV3, and pHV4) was in the majority (76% of all cases) TAC (Table S2).

### Acquisition of spacers from the targeting site

**Figure 6.**
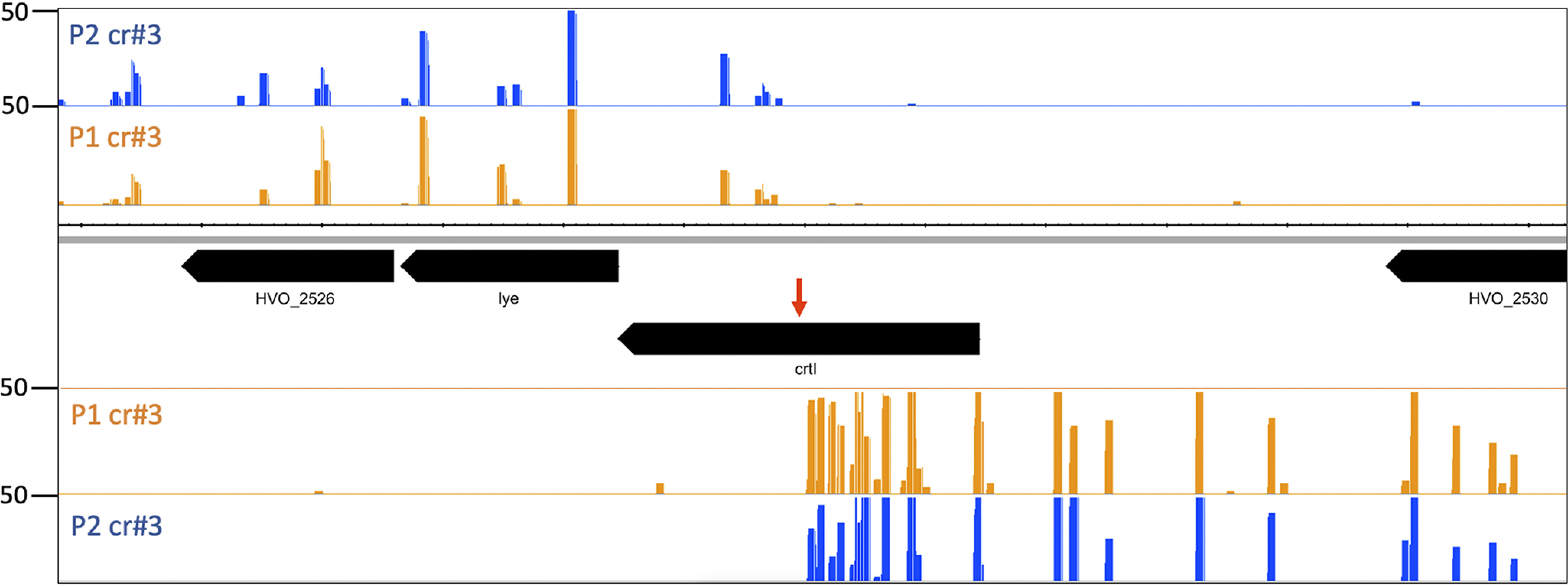
**Origin of new spacers from the targeting region.** Targeting the template strand of the *crtI* gene with crtI#3. Next-generation sequencing of new spacers in locus P1 and P2 showed that upon targeting the template strand many spacers originate from the vicinity of the targeting site (*red arrow*). Interestingly, more spacers originate upstream of the targeting site from the coding strand whereas fewer spacers are derived from the template strand. Number of spacer reads are shown at the *left*.

**Figure 7.**
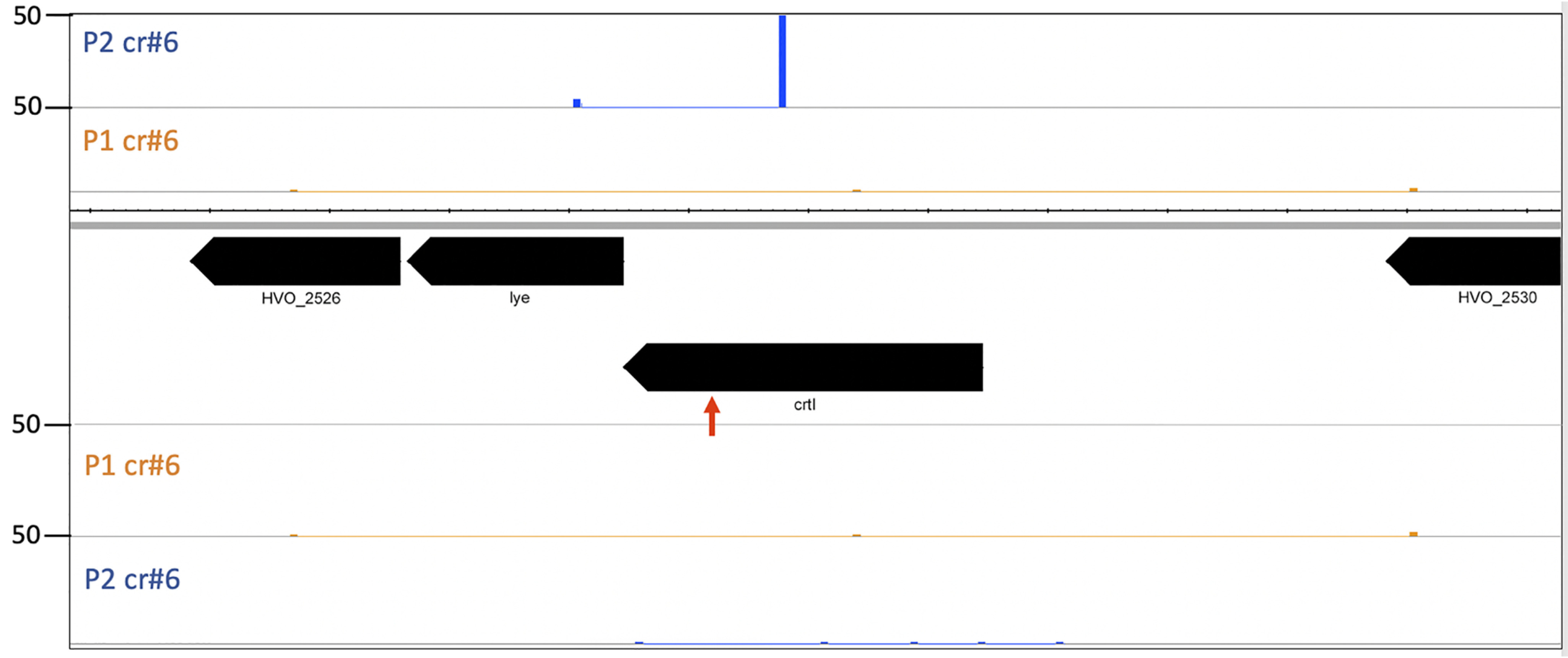
**Origin of new spacers upon targeting the coding strand with crtl#6.** Next generation sequencing of new spacers in locus P1 and P2 showed that upon targeting the coding strand only one major acquisition site is found originating from the template strand in the vicinity of the targeting site (*red arrow*). Number of spacer reads are shown at the *left*.

Analysis of newly integrated spacers with regard to the targeting site in the *crtI* gene showed that new spacers also originate from the vicinity of the targeting region (Figs. 6 and 7). Targeting the template strand with crRNA crtI#3 shows a hotspot at this site (Fig. 4) and leads to integration of a large number of spacers acquired from the coding strand upstream of the targeting site (Fig. 6), almost all spacers downstream of the targeting site were acquired from the template strand (Fig. 6). Additionally, the number of acquired spacers decreases with increasing distance to the targeting site. When targeting the coding strand with crRNA crtI#6 a single hotspot was observed with ∼150 reads close to the initial cleavage site of Cas3, located only on the template strand and only in locus P2 but not in P1 (Fig. 7). In contrast to loci P1 and P2, no new spacers were integrated into locus C. Locus C is located on the main chromosome, whereas loci P1 and P2 are encoded on the small chromosome pHV4 flanking the *cas* gene cassette, thus the different adaptation activities might be because of the location of the array. Alternatively, the difference in the leader sequence of the loci might be the reason for this difference in acquisition. The leaders of P1 and P2 share 95% sequence similarity, whereas the leader of locus C is only 75% identical to those of P1 and P2 (Fig. S4) (33).

### Adaptation under strong self-targeting conditions

**Figure 8.**
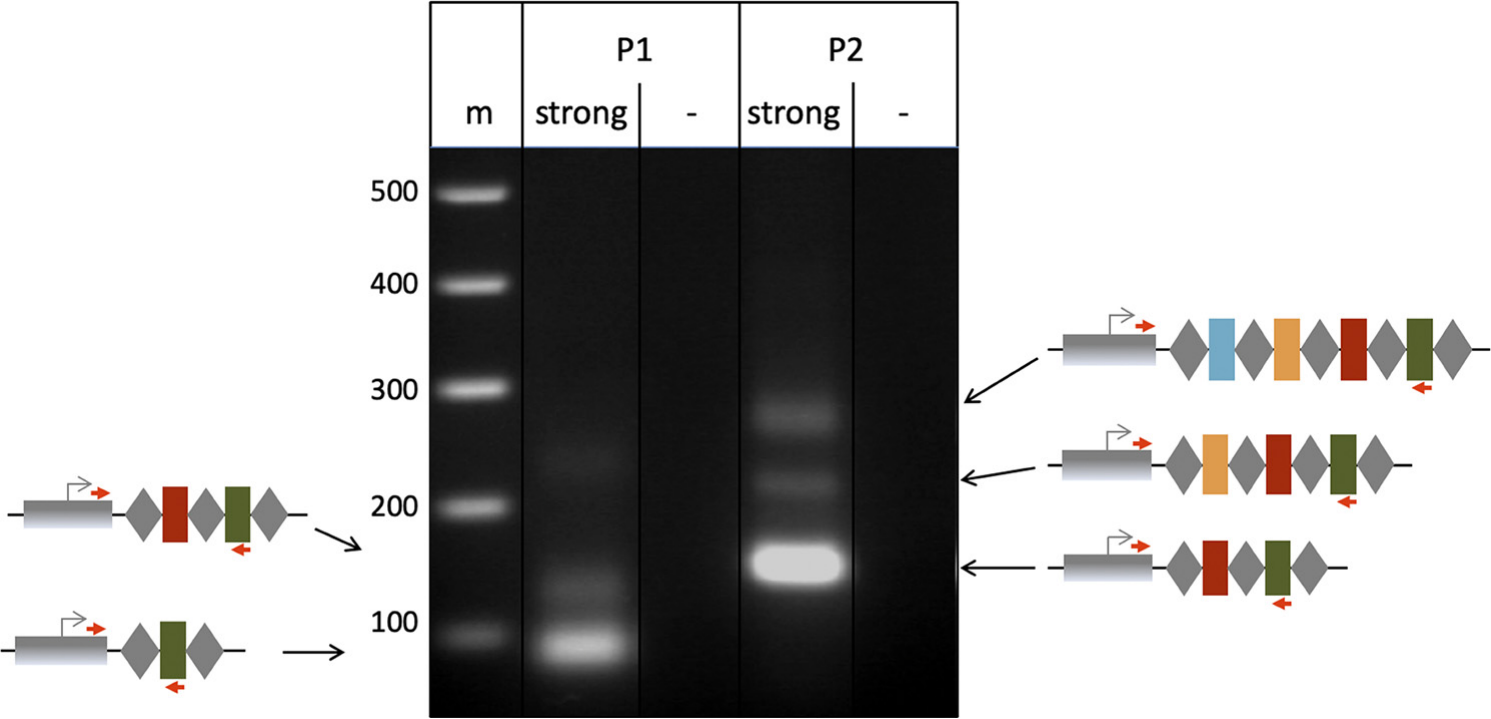
**Adaptation upon strong self-targeting.** PCR was used to amplify the 5′ end of loci P1 and P2. For P1, a longer product is visible upon induction of a strong self-targeting reaction (*lane strong*). For P2, products corresponding to one as well as two new spacer-repeat arrays (indicated by *orange* and *blue* spacers) are present (*lane strong*). *Lanes* −: control reaction without addition of template DNA; *m*: DNA size marker; sizes in bp are shown on the *left*. Schemes for expansion of loci are shown at the sides.

**Figure 9.**
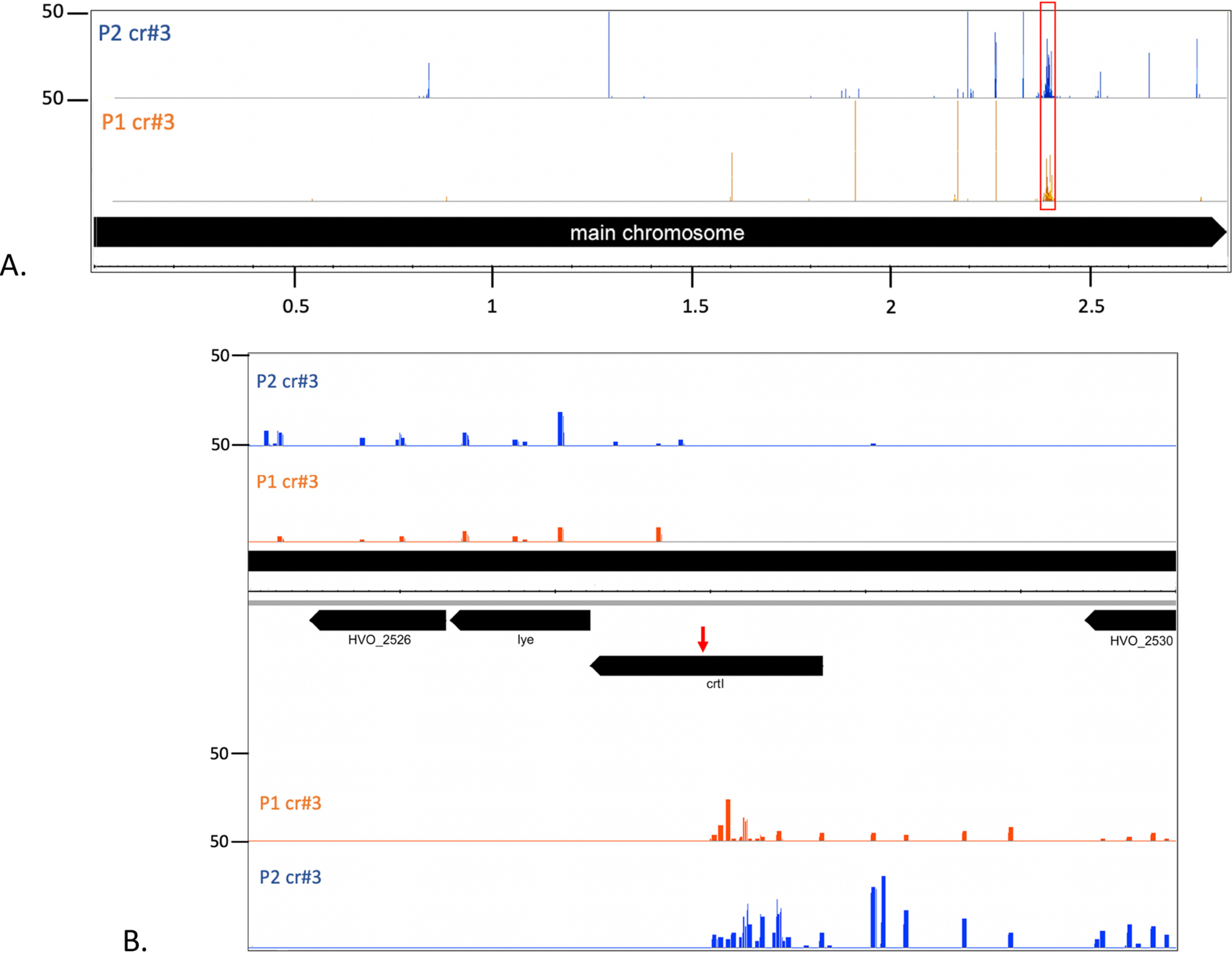
**Self-targeting with high concentrations of crRNA.** *A*, overview of acquisition from the main chromosome upon targeting with high concentrations of crRNA (from plasmid pTA232). A major hotspot is located at the targeting site (cluster of signals at 2.4 MB). The crRNA targeting site is indicated with a *red box*. *B*, acquisition from the vicinity of the targeting site. *Panels CHR*: annotation, chromosome coordinates are shown in the middle; *panel P1 cr#3*: spacers integrated in locus P1 upon targeting with crRNA crtI#3; *panel P2 cr#3*: spacers integrated in locus P2 upon targeting with crRNA crtI#3. Number of spacer reads are shown at the *left*.

To investigate whether a strong targeting reaction can also elicit adaptation, the Δ*cas6b* strains were transformed with a high-copy plasmid encoding the self-targeting crRNA crtI#3 (pTA232-tele-crtl#3) resulting in expression of high concentrations of cellular crRNA (26). Previous experiments have shown that the majority of cells transformed with this plasmid rapidly introduce deletions into the genome surrounding the targeting site, indicating that this strong self-targeting reaction leads to cleavage of all present genome copies (26). To investigate adaptation, PCR on loci P1 and P2 was performed with chromosomal DNA from this strain as a template. Larger products indicating the acquisition of one, and in some cases, two new spacers were obtained (Fig. 8). To elucidate the origin of the newly integrated spacers, HTS was performed. The number of acquired spacers was lower than the number of spacers obtained under low concentrations of self-targeting crRNAs (Figs. 4 and 9*A*.) (approx. 11,000 reads at high crRNA concentrations *versus* approx. 50,000 reads at low crRNA concentrations). Among the spacers acquired, the most distinct hotspot was located at the self-targeting site (Fig. 9*B*). The majority of the other peaks are located at hotspots previously found in the experiment using a weak self-targeting reaction.

### DNA fragments generated by endonucleases are not preferred sources for acquisition

Next we wanted to investigate whether DNA fragments generated *in vivo* by endonucleases are used as spacer precursors. For that purpose, we overexpressed the *mrr* gene (HVO_0682), which encodes a type IV restriction endonuclease (34, 35) in a Δ*cas6b* strain that was additionally transformed with a large amount (approx. 80 µg) of the plasmid pTA409. This plasmid has a methylation pattern (5′-G^me^ATC-3′) that is recognized by the restriction endonuclease Mrr, leading to DNA cleavage and thus providing potential spacer substrates. The genomic DNA of *Haloferax* is not methylated at these sequences (GATC) and is therefore not a substrate for the Mrr endonuclease. Successful expression of soluble Mrr endonuclease was confirmed using a Western blot (Fig. S5*A*). Spacer integration was investigated by PCR amplification of the 5′ end of locus P1. No products corresponding to an expanded locus could be obtained, which suggests that no spacer acquisition had taken place.

An additional form of double strand break can be generated in *Haloferax* by the homing endonuclease (HEN) encoded within the *polB* (HVO_0858) intein (36). Any DNA molecule that contains the target site, such as a plasmid (37), is therefore expected to be cut by the HEN. To test whether HEN activity is likely to generate substantial spacer acquisition via naïve adaptation we transformed *Haloferax* cells with plasmid pRL3, containing the HEN target site (36). This was followed by subsequent amplification of the 5′ ends of CRISPR locus P1 and P2 and sequencing of the obtained PCR fragments. Less than 1% of spacers originated from pRL3, only one spacer was derived from the HEN site itself, and 9 other spacers were acquired from DNA that was between 121 to 214 bases from the HEN target site (Fig. S5*B*). The overall low levels of spacer acquisition and relatively small number of spacers that originated from DNA proximal to the HEN target indicate that double strand breaks generated by HENs do not generate strong hotspots for spacer acquisition for the I-B CRISPR-Cas system of *H. volcanii*.

### The Δcas6b strain is active in naïve adaptation

**Figure 10.**
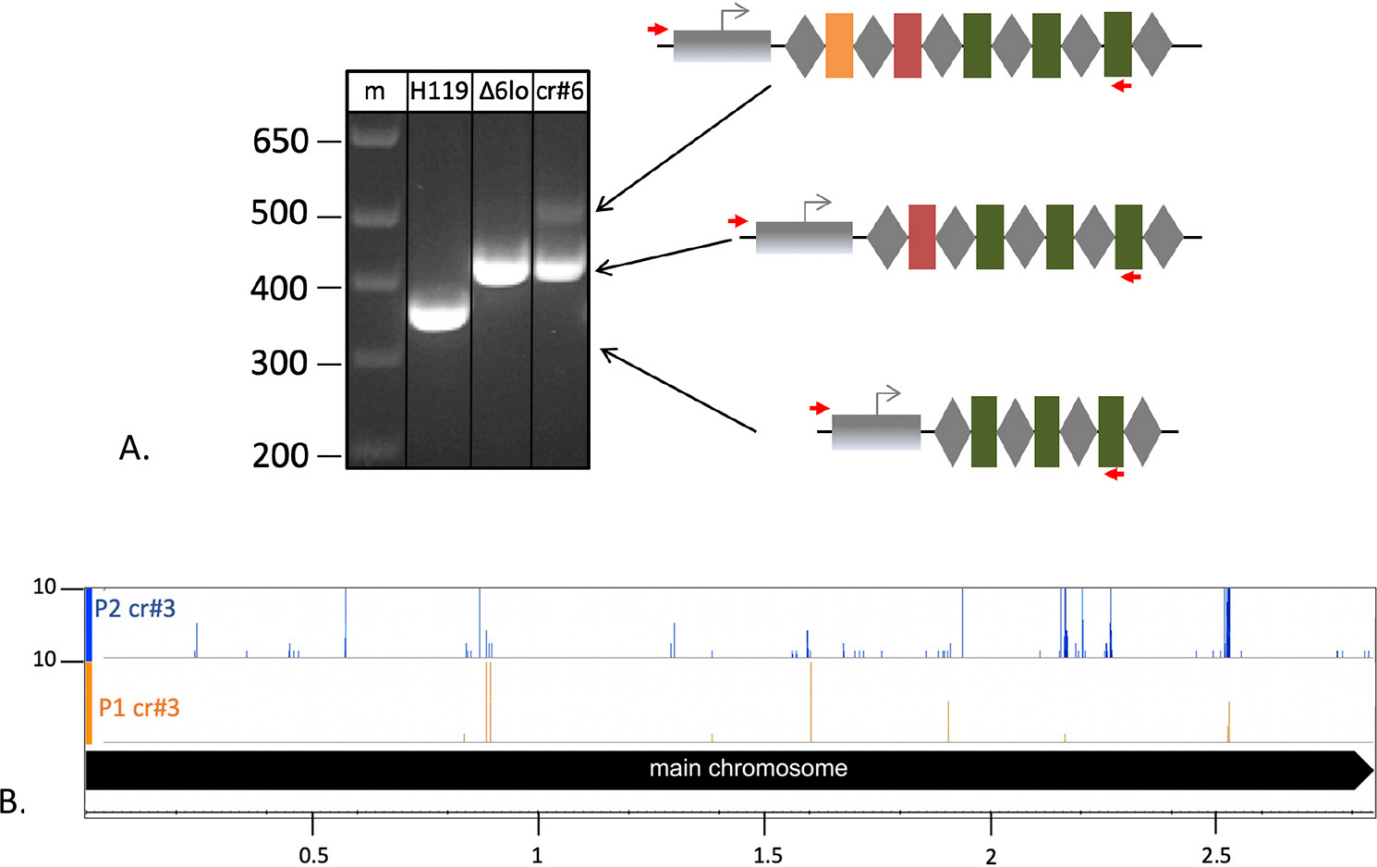
**Naïve adaptation in a Δ*cas6b* strain.** *A*, acquisition of new spacers into locus P1. PCR was performed to amplify the 5′ end of locus P1 using genomic DNA from one of the three strains: WT (*lane H119*), Δ*cas6blong* (*lane* Δ*6lo*) and Δ*cas6b* expressing crtl#6 from a high copy plasmid (*lane crtl#6*) as template. A product corresponding to an expanded locus (indicated by a *red* spacer) was not visible in the WT strain but was visible in the Δ*cas6b*long strain that was cultivated for a prolonged period. A Δ*cas6b* strain expressing crtl#6 from a high-copy plasmid (crtI#6) and therefore exhibiting targeting-induced adaptation served as a positive control. *Lane* −: negative control without the addition of genomic DNA. *B*, spacer origin overview. Newly obtained spacer sequences from locus P1 and P2 from Δ*cas6b*long were mapped to the genome. *Panels CHR*: annotation, chromosome coordinates are shown in the middle; *panel P1*: spacers integrated in locus P1; *panel P2*: spacers integrated in locus P2. Number of spacer reads are shown at the *left*.

We observed during our tests for spacer integration that a Δ*cas6b* strain that was grown in liquid culture for more than 3 months acquired new spacers spontaneously (Fig. 10*A*); we termed this strain Δ*cas6b*long. To elucidate the origin of these spacers obtained by naïve adaptation in the Δ*cas6b*long strain, we analyzed the PCR products from the extended loci using HTS. Spacers originating from the main chromosome clustered at a few hotspots (Fig. 10*B*). These hotspots are partly similar to the hotspots observed upon self-targeting with low concentrations of crRNA described above; they were also located at transposons or at highly transcribed genes (Fig. S6 and Table S3). These results indicate that the adaptation machinery is active to a low level in *Haloferax* in the absence of self-targeting, revealing naïve adaptation activity in this archaeon.

### Naïve adaptation induced in the WT strain

**Figure 11.**
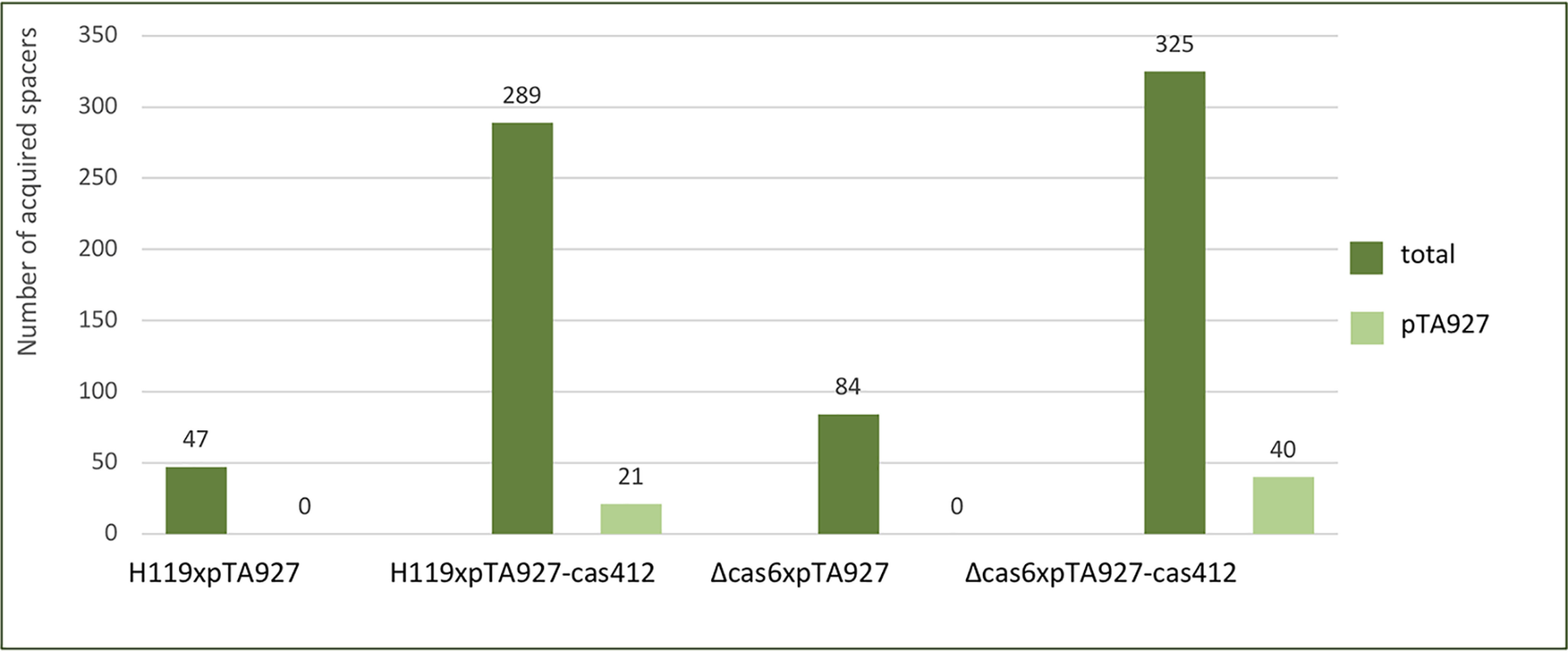
**Naïve adaptation in a WT strain.** In a WT *Haloferax* strain (H119xpTA927) naïve adaptation is inefficient and only few spacers are acquired, none of them from the plasmid pTA927. Upon overexpression of the adaptation proteins Cas1, Cas2, Cas4 in a WT strain (H119xpTA927-cas412) more acquisition can be observed with some spacers originating from the plasmid. The Δ*cas6b* strain acquires spacers from the chromosome but not from the plasmid (Δcas6xpTA927). However, upon overexpression of Cas1, Cas2, Cas4 more spacers are acquired in this strain including some from the plasmid (Δcas6xpTA927-cas412). Spacer integration into locus P2 is shown (see also Table S4). *Dark green* (total): spacers from all chromosome and the plasmid pTA927; *light green* (pTA927) spacers only from pTA927.

To investigate whether naïve adaptation is more active upon overexpression of the adaptation proteins Cas1, Cas2, and Cas4, WT strain H119 as well as the Δ*cas6b*long strain were transformed with either the plasmid pTA927 (without insert) or pTA927 expressing Cas1, Cas2, and Cas4. PCR was performed on locus P2 to test for adaptation, but larger PCR products corresponding to an expanded locus could not be observed. Nevertheless, DNA from gel regions corresponding to the expected size range was extracted, cloned, and HTS was carried out. The number of new spacers acquired overall was very low, but in both strains additional expression of *cas1*, *cas2*, and *cas4* resulted in 4- to 6-fold more spacers, as compared with the control (with pTA927 without insert); spacers also originated from the pTA927 plasmid (Fig. 11 and Table S4). However, no consensus PAM and no clear acquisition hotspots or strand preference could be identified.

## Discussion

In recent years, the mechanisms underlying adaptation have been elucidated in a number of CRISPR-Cas systems. Until now only a single study has reported spacer acquisition in *H. volcanii*, and in that case the process was seemingly activated by mating with a different species (33). Mating in haloarchaea involves cytoplasmic bridges and allows one mating partner to cleave the genome of the other via endonucleases (37), and therefore could induce adaptation. In this study, we triggered adaptation in *H. volcanii* by CRISPR-Cas self-targeting, which causes DNA breaks, to investigate the resulting spacer integration.

### Self-targeting–induced adaptation occurs in the H. volcanii type I-B system

Our previous experiments have indicated that an interference reaction against genomic DNA is only moderately toxic in *H. volcanii* and results in genomic deletions surrounding the targeted locus (26). The DNA fragments generated during this process are potential prespacer substrates for the adaptation machinery as was recently shown for type I-E and I-F systems (39). For type I-F, I-E, and I-B systems, a model has been postulated in which cleavage products generated by Cas3 are captured and utilized by the adaptation complex as spacer substrates (13, 16–18, 39, 40). Indeed, we could demonstrate here acquisition of new spacers into the CRISPR loci P1 and P2, but not locus C, under limited self-targeting conditions. This is in agreement with a previous study (33), however, the reason for the inactivity in spacer acquisition of locus C remains unknown. Because the leader sequences of loci P1 and P2 share a sequence similarity of 95%, and the C leader is only 75% identical to P1/P2 (Fig. S4), it is reasonable to speculate that one or several mutations in the leader prevent adaptation. A study in *Haloarcula hispanica* indeed showed that the leader sequence is important for adaptation (41).

### Self-targeting–induced adaptation sources the genome for spacer templates

All new spacers found in loci P1 and P2 upon self-targeting originated from genomic DNA (main chromosome and chromosomal plasmids), indicating that no mechanism exists in *H. volcanii* to prevent the acquisition of self-targeting spacers. This is in line with a previous study on mating of *Haloferax* species, showing that for the *Haloferax mediterranei* type I-B system a greater number of spacers was acquired from self-replicons than from the mating partner's genome (33). For the type I-E system of *Escherichia coli*, a strong bias for acquisition plasmid over chromosomal DNA has been shown. This can be ascribed to the high frequency of Chi sites in the *E. coli* genome, serving as attenuation signals for the DNA resection complex RecBCD, thus preventing excessive production of genomic DNA fragments that could be used as spacer precursors (42). The *Haloferax* genome does not contain Chi sites or (to the best of our knowledge) any similar nuclease attenuation signals, which might explain the efficient acquisition of chromosomal spacers observed here. Studies with other CRISPR-Cas systems, such as the type II systems of *Streptococcus thermophilus* and some Bifidobacteria, as well as the type I-F system of *Pectobacterium atrosepticum*, have also shown the acquisition of genomic spacers (17, 19, 21). This indicates that self-acquisition is not inhibited in these organisms, but because of its usually cytotoxic effects (17, 23, 24) it is rarely observed. The strain used in our experiments is deleted for *cas6b*; therefore the newly acquired self-targeting spacers are not expressed as mature crRNAs. Additionally, our previous studies have shown that a self-targeting crRNA is in fact tolerated extremely well in a WT strain that additionally contains 51 endogenous crRNAs (26). Nevertheless, if such spacers accumulated, the deleterious effects of self-targeting might be manifested more strongly. Therefore, it has been suggested that the reason that expression of the adaptation machinery is repressed under standard conditions is to prevent random acquisition from the genome (33).

We observed TAC as a PAM sequence for adaptation for the majority of the spacers indicating a much stricter PAM recognition mechanism for adaptation than for interference, which tolerates seven different motifs (29, 33). Utilizing self-targeting to induce adaptation, we observed spacer acquisition from the main chromosome as well as from the small chromosomes, with largely identical hotspots for spacers integrated into loci P1 and P2 and irrespective of which targeting crRNA was expressed. The vast majority of these hotspots are located at transposase genes, whereas some span highly transcribed genomic regions like the rRNA genes. This might indicate that spacers are increasingly acquired from regions with a higher occurrence of free DNA ends, namely sites with transposon activity or frequent double-strand breaks caused by stalling of DNA replication forks because of collision with RNA polymerase.

We also found hotspots at the *orc11* and *orc14* genes that are part of an integrated provirus region that is AT rich compared with the standard GC rich sequence found in the *Haloferax* genome (35). Increased acquisition from the provirus implies that this element probably occasionally excises from the genome and potentially initiates replication, thus becoming a preferred target for spacer acquisition. A similar phenomenon of a spacer acquisition hotspot near a replicating proviral element was shown for an *H. mediterranei* provirus (33).

A similar adaptation pattern was observed for *Pyrococcus furiosus*, where hotspots localized at sites with an increased risk of DNA nicking or double-strand breaks, such as transposons, highly transcribed regions, or active CRISPR loci (43). In the study of adaptation during *Haloferax* mating, the majority of spacers in *H. volcanii* was also acquired from the vicinity of the two CRISPR loci P1 and P2 (33). However, we did not detect any spacer hotspots at these sites. This indicates that the mating process in which the plasmid encoding the actively growing array can move between cells may result in a different pattern of spacer acquisition than that following self-targeting.

HTS of CRISPR loci P1 and P2 upon strong self-targeting revealed the acquisition of approx. 11,000 spacers, about 20% of the number of spacers acquired under weak self-targeting conditions, with only one distinct hotspot at the targeting site. This is likely because the autoimmune reaction is abolished swiftly by deletion of the targeting site, resulting in a smaller number of cleavage products that could fuel spacer acquisition, and possibly arrest of adaptation. Similar to the weak targeting, we found for the strong targeting-induced adaptation TAC as the major PAM for adaptation.

### A closer look at adaptation in the targeting region reveals strand biases

Analysis of the targeting region in the *crtI* gene revealed that targeting the coding strand with crRNA crtI#6 expressed from a low-copy plasmid resulted in the acquisition of only a single spacer from the template strand upstream of the initial cleavage site. This might indicate an extremely weak self-targeting reaction, which would result in a low degree of Cas3 cleavage and consequently a small number of potential spacer precursors or free DNA ends for the adaptation machinery to utilize. Previous studies with crRNA targeting in the frame of a CRISPRi approach revealed clear differences between different crRNAs depending on their binding location (32, 44). Currently the parameters for an effective crRNA targeting are not known (32).

**Figure 12.**
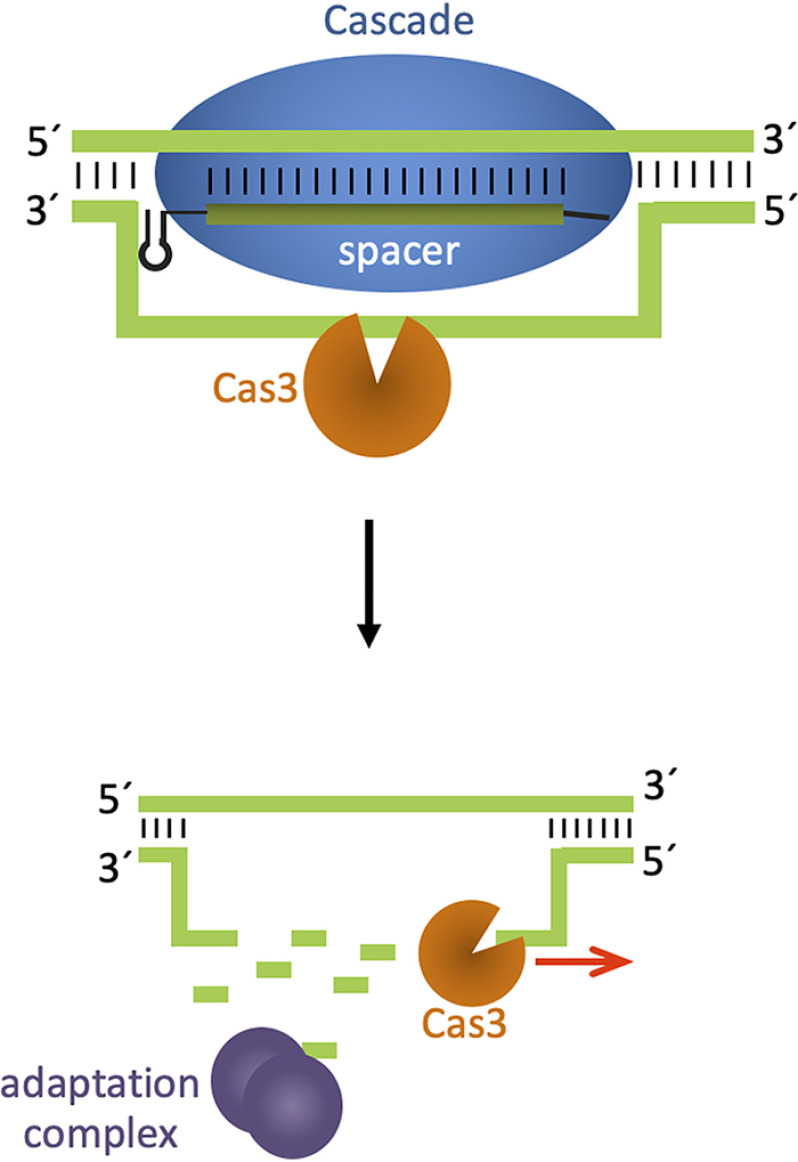
**Degradation of chromosomal DNA at the targeting site.** Initial cleavage of the genomic DNA is catalyzed by Cas3. The strand opposite to the crRNA targeting site is degraded by Cas3 in 3′–5′ direction, where Cas 3 unwinds and cleaves. However, the enzyme catalyzing cleavage of the strand, to which the crRNA binds, has not been identified.

Upon targeting of the template strand with crRNA crtI#3, a clear strand bias with respect to the target site was observed, with spacers upstream of the initial cleavage site being derived from the coding strand and downstream spacers originating from the template strand. Additionally, the number of acquired spacers decreases with increasing distance to the targeting site. The type I-E system of *E. coli* displays a bias during primed adaptation for acquisition from the same strand as the priming protospacer (12, 18); a similar adaptation pattern as the one reported here for *Haloferax* has been observed in the I-B system of *Haloarcula hispanica* and in type I-F systems (13, 15, 17). Furthermore, our data hint at the mechanism of action of Cas3, because the strand bias suggests a model similar to what has been proposed for other type I systems (13, 45–47): Cas3 initially cuts the strand displaced by Cascade and subsequently unwinds and cleaves the strand in a 3′–5′ direction, thereby generating DNA fragments that the adaptation machinery can utilize as spacer substrates (Fig. 12) (17, 40). Acquisition of spacers upstream of the targeting site might be because of Cas3 flipping onto the other DNA strand and proceeding to unwind and cleave it in a 3′–5′ direction, as has been suggested for *H. hispanica* (13). However, it is also possible that DNases from other DNA repair or recombination pathways are involved in cleaving the DNA, because more and more reports show the involvement of host enzymes in CRISPR-Cas reactions (48, 49); this remains to be elucidated.

### The H. volcanii WT strain is capable of low-level basal naïve adaptation

During our experiments, we have observed that a Δ*cas6b* strain cultivated for an extended period of time (at least 3 months) (Δ*cas6b*long) exhibits naïve adaptation and acquires spacers from genomic DNA, similar to a *Sulfolbus islandicus* Δ*cas6b* strain displaying increased adaptation frequency (50). Such strains do not suffer a fitness cost from CRISPR targeting following adaptation, because due to the deletion of the *cas6b* gene the pre-crRNAs cannot be processed and thus no targeting can take place, leaving them free to accumulate spacers. Using HTS, we could detect approx. 2600 new spacers in loci P1 and P2 of the Δ*cas6b*long, a much smaller number than we obtained for targeting-induced adaptation. Furthermore, spacer hotspots were less pronounced and not located at transposons or highly transcribed genes. These results indicate that naïve adaptation in the *cas6b* deletion strain is an inefficient random process, which is in line with observations in *E. coli* or *P. atrosepticum*, where primed adaptation is vastly prevalent, or *H. hispanica*, where only primed adaptation has been observed thus far (13, 18, 51). The fact that parts of the hotspots were located at different sites scattered across the genome might indicate that a differing mechanism underlies naïve adaptation compared with targeting-induced adaptation.

Overexpression of adaptation proteins increased naïve adaptation in Δ*cas6b* as well as in the WT strain, with a slightly higher number in total for the Δ*cas6b* strain. This suggests that inefficient naïve adaptation is also possible in a WT strain, and its frequency can be increased with enhanced concentrations of the adaptation machinery, in line with the fact that the adaptation proteins are not expressed under standard growth conditions (30). Thus, the regulation of Cas1, Cas2, and Cas4 might prevent random acquisition of spacers from the own genome.

In both strains, no adaptation hotspots or consensus PAM could be identified, indicating a somewhat random and undirected adaptation mechanism. This might be because of the additional expression of the adaptation machinery, similar to the decrease of spacers with a consensus PAM upon up-regulation of *cas1*, *cas2*, and *cas4* in *S. islandicus* (52). A small percentage of spacers were also acquired from the plasmid expressing the genes required for adaptation, showing that it can be a source for naïve adaptation as well. The Δ*cas6b* strain was more active in integrating fragments of its own genome than a WT strain, suggesting that although it is not lethal for a WT strain to integrate fragments of the own genome, it might nevertheless reduce cell fitness to some extent.

### Adaptation in archaeal type I-B systems

Although type I-B systems are the most common CRISPR-Cas systems and present in both bacteria and archaea, to date adaptation in type I-B system has been studied *in vivo* in only two other archaeal organisms: *H. hispanica* and *P. furiosus*. For *H. hispanica* only primed adaptation has been reported (13) whereas in *P. furiosus*, primed as well as naïve adaptation have been recently observed (43, 53). Curiously, in *P. furiosus* naïve adaptation partially depended on the interference machinery, which is known as strict prerequisite for primed adaptation. Although *H. volcanii* and *H. hispanica* are somewhat related haloarchaea their I-B systems are highly divergent, *e.g.* their PAM preferences differ (54) and the Cas1 proteins share only 56% identity. It is therefore unsurprising that we show here that in contrast to *H. hispanica* naïve adaptation is active in *H. volcanii.*

### Different types of acquisition: Naïve, targeted, and primed

During naïve acquisition invaders that have previously not been encountered are recognized and pieces of their DNA are used as prespacers and integrated into the CRISPR loci. This process requires only the adaptation proteins. In contrast, primed adaptation is triggered by a mismatching crRNA or nonfunctional PAM and requires the complete interference machinery (for type I-B: Cascade with crRNA and Cas3) as well as the adaptation proteins (Cas1, Cas2, Cas4). Staals *et al*. (17) have shown a third type of acquisition: Targeting acquisition. Here, the standard defense reaction where a matching crRNA binds perfectly to the invader triggers acquisition alongside the degradation of the targeted DNA. This interference-coupled priming process may actually be the dominant mode of primed adaptation, but may sometimes be more difficult to detect, if targets are too rapidly destroyed, as in the case of small plasmids. The self-targeting reaction described here represents a form of targeting acquisition, because a perfectly matching crRNA triggers efficient adaptation, and because the target is the chromosome.

## Conclusion

Taken together, we have shown that self-targeting leads to the acquisition of spacers from genomic DNA. The autoimmune reaction in *H. volcanii* can therefore, similar to mating, induce adaptation. Presumably, DNA damage is sensed and expression of the adaptation proteins is induced, resulting in capture of DNA fragments generated by the interference reaction, as well as from sites with an excess of free DNA ends. Our results indicate that the adaptation machinery acquires spacers from sites with frequently exposed DNA ends, such as the targeting site, transposases, and highly transcribed regions. The acquisition of DNA fragments from transposable elements could be a means to prevent hyperexpansion of these elements, which could be detrimental for the cell.

Interestingly, the extent and pattern of adaptation resulting from self-targeting strongly depends on the strength of self-immunity pressure imposed. As observed for other systems, adaptation at the targeted site exhibits a strand-specific biased pattern that is probably dictated by the mechanism of action of Cas3. Furthermore, naïve basal adaptation is possible in *H. volcanii* but is exceedingly inefficient. Overexpression of the adaptation genes results in PAM-independent acquisition.

## Experimental Procedures

Strains, plasmids, and oligonucleotides used are listed in Tables S5–S7.

### Strains and culture conditions

*H. volcanii* strains H119, Δ*cas6b* (HV30), HV32, and HV35 were grown aerobically at 45°C in Hv-YPC medium (56, 57). Strains with plasmids were grown in Hv-Ca or Hv-min medium with the appropriate supplements. *E. coli* strains DH5α and GM121 were grown aerobically at 37°C in 2YT medium.

### Construction of plasmids

The construct containing a crRNA targeting the coding strand of *crtI* was generated by inverse PCR with pMA-RQ-telecrRNA as template using the primers crtI#6iPCRup and crtI#6iPCRdo. Primers omit the original spacer and contain the new spacer sequence. The resulting plasmid contains a synthetic *Haloferax* promoter, the crRNA flanked by t-elements, and a synthetic *Haloferax* terminator.^6^ The insert was excised from the plasmid with BamHI and KpnI and ligated into the shuttle vector pTA232, resulting in the plasmid pTA232-crtI#6.

For generation of plasmid pTA927-p.tnaA-cas4-1-2, the *cas4*, *cas1*, and *cas2* genes were amplified using the primers Hindcas4Start and cas2StoppBamHI. The PCR product was purified, digested with HindIII and BamHI, and subsequently ligated with a pTA927-p.tnaA vector digested with the same enzymes, resulting in the plasmid pTA927-p.tnaA-cas4-2-1.

Plasmid pTA232-p.fdx-mrr and pTA231-p.fdx-mrr were generated as follows. The gene for the endonuclease Mrr (HVO_0682) was amplified from *H. volcanii* genomic DNA using primers 5′-mrr-NdeI and 3′-mrr-XbaI, the resulting fragment was ligated with the linearized pBluescript vector (digested with *EcoR*V), resulting in pBlue-5′mrr-NdeI. The *mrr* gene was isolated from this plasmid by digestion with NdeI and XbaI and ligated into pTA232-p.fdx and pTA231-p.fdx (both digested with NdeI and XbaI), resulting in pTA232-p.fdx-mrr and pTA231-p.fdx-mrr. To generate pTA231-p.fdx-mrr-NFLAG the gene for the endonuclease Mrr was amplified from *H. volcanii* genomic DNA using primers 5′-mrr-*SnaB*I and 3′-mrr-XbaI. The resulting fragment was ligated with the linearized pBluescript vector (digested with *EcoR*V), resulting in pBlue-5′mrr-*SnaB*I. The *mrr* gene was isolated from this plasmid by digestion with *SnaB*I and XbaI and ligated into pTA231-p.fdx-NFLAG (digested with *SnaB*I and XbaI), resulting in pTA231-p.fdx-mrr-NFLAG.

### Preparation of samples for next generation sequencing

For sequencing of amplicons to identify acquired spacers, *H. volcanii* cells were transformed with the respective plasmids using the previously described PEG method (34). All used plasmids were passaged through *E. coli* strain GM121 to avoid methylation. The Δ*cas6b* strain was transformed with a plasmid encoding a crRNA targeting either the coding strand or the template strand of the *crtI* gene, and cultures were inoculated directly after transformation. A culture with an OD_650_ of approx. 0.2 was harvested and genomic DNA was isolated. The guide DNA served as template for a PCR amplifying the 5′end of each CRISPR locus, spanning part of the leader and the 5′ end of the first, second, or third spacer of each locus (primers see Table S7). PCR products corresponding to a locus expanded by one new repeat spacer unit were isolated from an agarose gel and served as templates for a PCR adding adapter sequences to the amplicons. Used forward primers (P1-fw for locus P1, P2-fw for locus P2) could bind to the 3′ end of each leader and contained the forward adapter sequence, whereas reverse primers (P1.1-rev for locus P1, P2.1-rev for locus P2) could bind to the 5′ end of the first spacer of each locus and contained the reverse adapter sequence. To add diversity to the amplicons, used primers contained 0–3 random bases between the adapter sequence and the locus-specific sequence. After isolation of the PCR products from an agarose gel the products were used as template for a PCR adding index sequences, using primers from the Nextera Index Kit (Illumina). The size of obtained products was checked with a QIAxcel Advanced Instrument electrophoresis device (Qiagen), and they were purified using Agencourt AMPure XP magnetic beads (Beckman Coulter). After quantification of the purified products using a Qubit fluorometer and the Qubit dsDNA BR Assay Kit (Invitrogen), all samples were diluted to a concentration of 4 nm. Dilutions were pooled and used for the sequencing reaction, which was carried out on a MiSeq sequencing device (Illumina) using the MiSeq^®^ Reagent Nano Kit v2 (Illumina) according to the manufacturer's instructions.

### Acquisition upon mrr endonuclease overexpression and Western blot analysis

*Haloferax* cells (HV50) were transformed with pTA232-p.fdx-mrr. Guide DNA was isolated and all three CRISPR loci were investigated for newly integrated spacers as described above. To confirm the soluble expression of the Mrr endonuclease, *Haloferax* cells (HV50) were transformed with pTA232-p.fdx-mrr-NFLAG and grown to stationary phase. A soluble protein extract was isolated and proteins were separated on a 10% SDS-PAGE and subsequently transferred to a membrane. The membrane was incubated with antibodies against the FLAG tag.

### Acquisition following cutting by the endogenous HEN endonuclease

*H. volcanii* cells (WR536) were transformed with pRL3 (36). This plasmid contains the site cleaved by the HEN endonuclease with short (250 bases on each side) flanking regions of the *polB* intein. Colonies were screened by PCR using primers IS-124 and IS-125 followed by agarose gel electrophoresis of the products. About 45% of the colonies had an amplification product that was unchanged, whereas 55% were shortened in the region of interest, indicating that the HEN had cut the plasmid, but that invasion had not been completed, and it was repaired via a pathway other than homologous recombination. The transformation protocol was repeated twice, 200 colonies per biological replicate were scraped off, and DNA was extracted and amplified as described (33). The amplicons underwent size selection and Illumina sequencing and were analyzed for spacer match location as described previously (33).

### Bioinformatics of analysis of HTS data and identification PAM

Sequenced reads in the FASTQ files were inspected using the FastQC tool (58), then the TrimGalore tool (RRID:SCR_011847) was used to remove adapters and perform read quality trimming. The reads were then aligned to the main chromosome and the five plasmids via Bowtie2 (55). After that, an in-house python script was used to investigate if new spacers were integrated into the CRISPR array. Once new spacers were determined, we used Bowtie2 to map those to the reference genomes, which are now called protospacer sequences. For each protospacer, we extracted length, position, and strand. Finally, the PAM motif in the DNA upstream of the protospacer positions were obtained for protospacers with at least 10 reads via the WebLogo tool (38).

## Data availability

The sequences obtained have been deposited in the European Nucleotide Archive (ENA) with the primary accession number PRJEB39506. All remaining data are contained within the article.

## Supplementary Material

Supporting Information

## Footnotes

6 A. Sabag-Daigle and C. J. Daniels, in preparation.
