# Supplementary material for: Adaptation induced by self-targeting in a type I-B CRISPR-Cas system
Source: J Biol Chem. 2020 Jul 28;295(39):13502–15. doi: 10.1074/jbc.RA120.014030 (PMC7521656; doi:10.1074/jbc.RA120.014030)
Supplement: Supporting Information [file supp_295_39_13502__index.html]

Adaptation induced by self-targeting in a type I-B CRISPR-Cas system — Self-targeting induced adaptation — Adaptation induced by self-targeting in a type I-B CRISPR-Cas system — Self-targeting induced adaptation — Supporting Information 

# Adaptation induced by self-targeting in a type I-B CRISPR-Cas system

## Supporting Information

- Supporting Information (to be published online) - Supplementary Figures and Tables
