# Supplementary material for: Adaptation induced by self-targeting in a type I-B CRISPR-Cas system
Source: J Biol Chem. 2020 Jul 28;295(39):13502–15. doi: 10.1074/jbc.RA120.014030 (PMC7521656; doi:10.1074/jbc.RA120.014030)
Supplement: Supporting Information [file supp_RA120.014030_160371_1_supp_559767_qcxtdv.pdf]

Supplementary Data

Supplementary Figures 1-6 page 1

Supplementary Tables 1-7 page 7

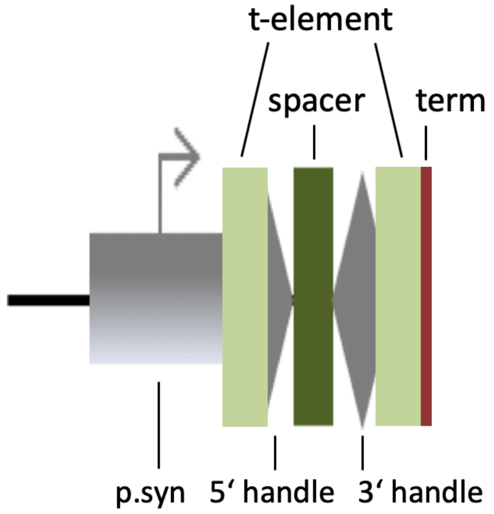

Supplementary Figure 1. The crRNA expression cassette. Expression is controlled by the strong constitutive promoter p.syn, the genes for the mature crRNA (containing a spacer, an 8 nt long 5‘ handle and a 22 nt long 3‘ handle is flanked by genes for t-elements.

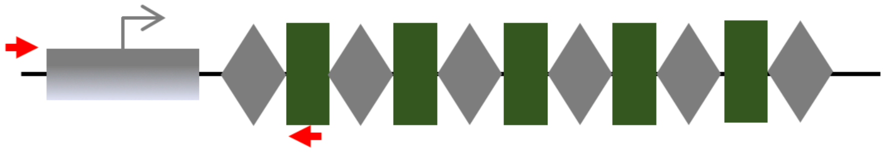

A.

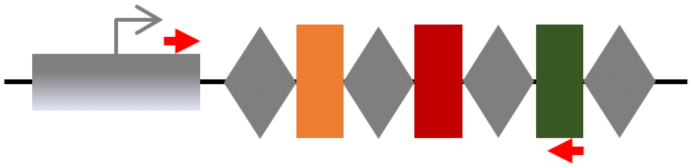

B.

Supplementary Figure 2. Analysis of adaptation events. Primers used to amplify the 5´ part of the CRISPR loci are shown as red arrows. Integration of new spacers were investigated by PCR using a primer binding upstream (A.) or in the leader region of the CRISPR locus (B.) and a primer binding to one of the spacers, for example the first one (A.) or the third one (B.).

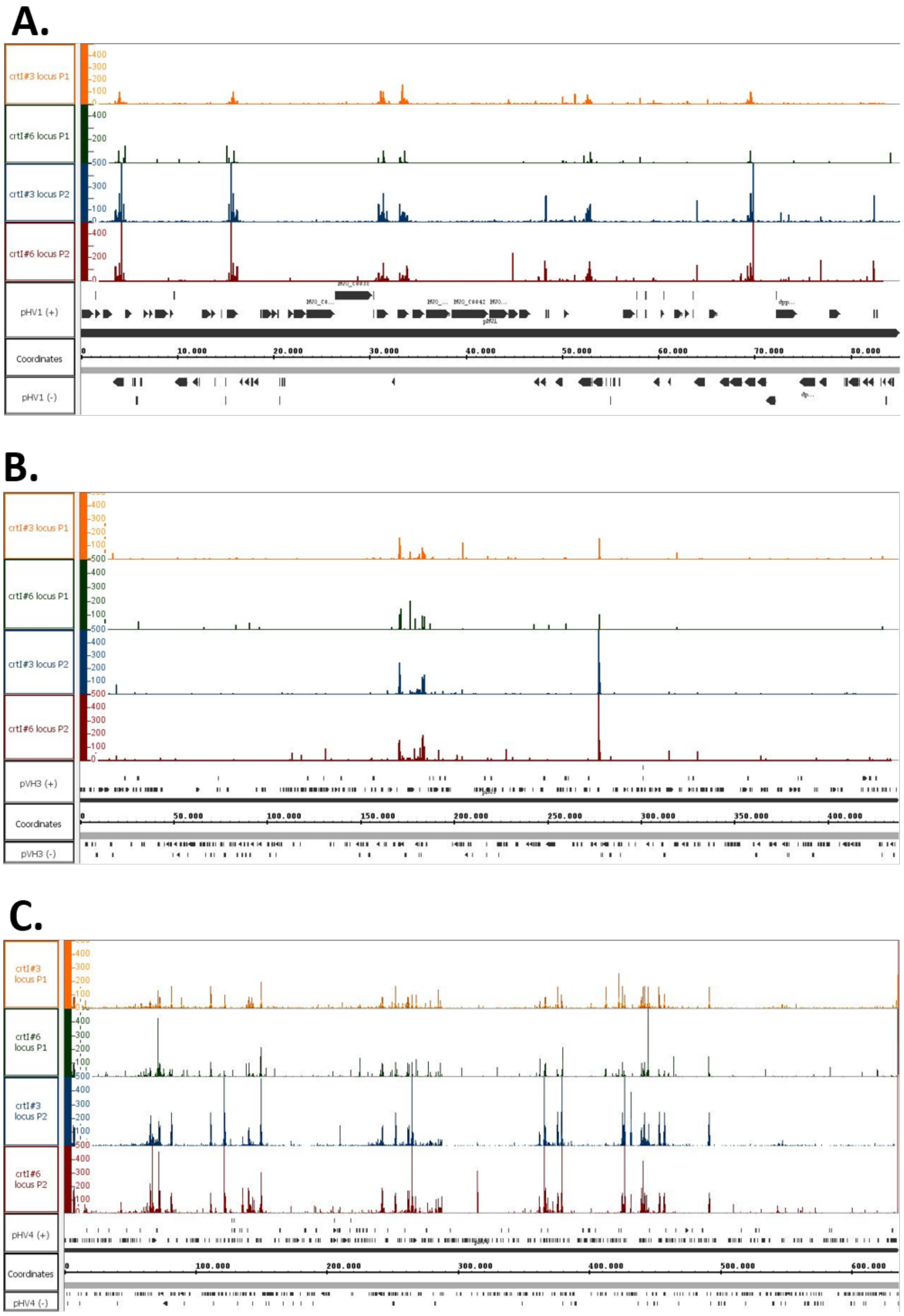

Supplementary Figure 3. New spacers originate also from chromosomal plasmids pHV1, pHV3 and pHV4. A. Acquisition from pHV1. Panel pHV1: annotation. **B. Acquisition from pHV3.** Panel pHV3: annotation. **C. Acquisition from pHV4**. Panel pHV4: annotation. Panel crtI#3locusP1: new spacers integrated in locus P1 upon targeting with crRNA crtI#3; panel crtI#6locusP1: new spacers integrated in locus P1 upon targeting with crRNA crtI#6; panel crtI#3locusP2: new spacers integrated in locus P2 upon targeting with crRNA crtI#3; panel crtI#6locusP2: new spacers integrated in locus P2 upon targeting with crRNA crtI#6.

```
P1      GTTTCCGTCGACCCTC-GGGGGGTACGGGGGAATTGAGGG  39
P2      ATTTCCGTCGACCCCCCGGGGGGTACAGGGGAATTGAGGG  40
C       GTTTTCGTCGACCCCCCGGGGGTTGCGGGTGAATTGAGGG  40

P1      TCGACGGAAACGTTGATGTGAGTTCGCTATGTACAGCCGT  79
P2      TCGACGGAAACGTTGATGTGAGTTCGCTATGTACAGCCGT  80
C       TCGACGGAAACACTCTTTTGAATTCGGGTGATACACATCA  80

P1      CTACCCCCGTGAATTCGGACGG  101
P2      CTACCCCCGTGAATTCGGACGG  102
C       TTGTACCCGTGAATTCGGGATG  102
```

Supplementary Figure 4. Sequence alignment of the CRISPR locus leaders of *H. volcanii*. Sequences from loci P1, P2 and C were aligned using the ClustalW server (Kyoto University Bioinformatics Center) and visualized with SnapGene Viewer (GSL Biotech LLC). The leader sequences of P1 and P2 are 95% identical to each other, whereas the C leader is only 75% identical to P1 and P2.

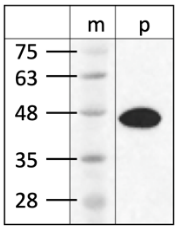

A.

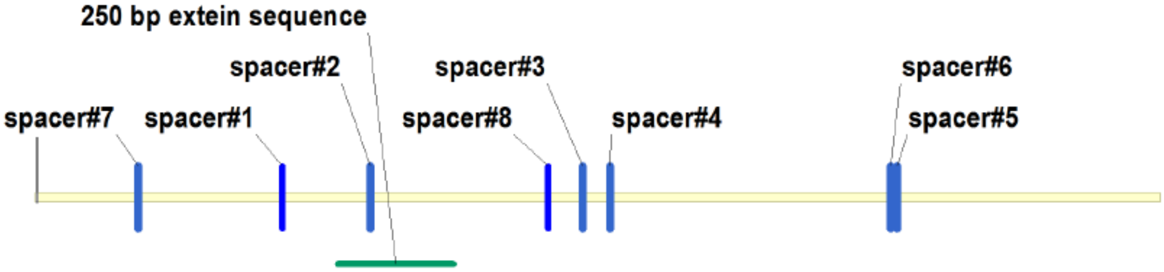

B.

Supplementary Figure 5. A. The endonuclease Mrr is expressed. To confirm that the Mrr endonuclease is solubly expressed in *Haloferax,* soluble protein extracts were isolated, proteins were separated on an SDS-PAGE and transferred to a membrane that was subsequently probed with a FLAG antibody. Lane m: protein size marker, sizes are given at the right in kDa, lane p: S18 extract. The theoretical molecular weight of the Mrr protein including the FLAG-tag is 39 kDa. Halophilic proteins migrate slower on SDS PAGE due to their negative charges. **B. Spacers acquired due to HEN cleavage.** Schematic representation of the pRL3 plasmid (5038 bp) and the location of the spacers originating from it. Each unique spacer sequence is numbered 1-8 with an indication of its location in bp from the beginning of the sequence. The target site (extein) is shown in green.

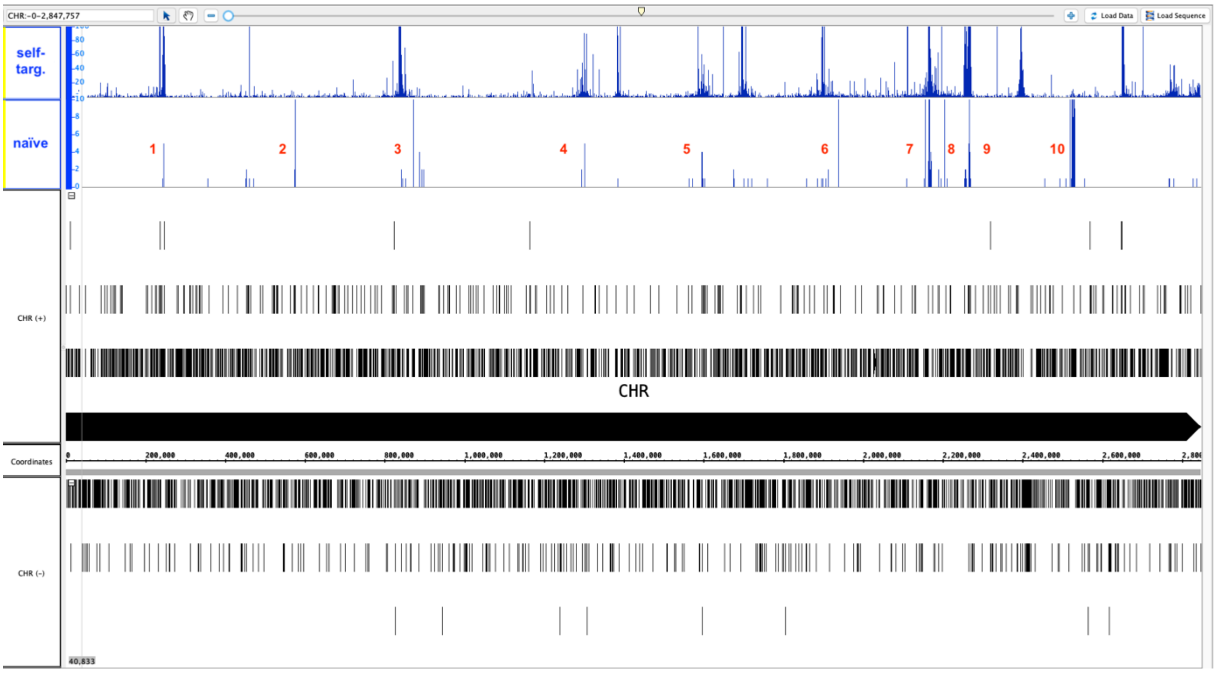

Supplementary Figure 6. Spacer origins for naïve and targeting induced adaptation. The hotspots of spacer acquisition upon self-targeting and naïve adaptation are partly identical but to some extent they also differ. Hotspots found for naïve adaptation were numbered and compared to hotspots found upon self-targeting, see Supplementary Table 2. Panels CHR: annotations, coordinates are shown in the middle; panel self-targ.: spacers acquired upon self-targeting; panel naïve: spacers acquired during naïve adaptation.

Supplementary Tables

Supplementary Table 1. Spacer acquisition from transposase genes. The number of unique spacers acquired from the genome (column total number unique spacers) and from transposase genes (column unique spacers transposase genes) is listed, the percentage of spacers acquired from transposase genes is listed in column %. Column locus/ crRNA shows into which locus the acquired spacers were inserted (locus P1 or P2) and which crRNA triggered the acquisition (cr#3 or cr#6).

locus/ crRNA	total number unique spacers	unique spacers transposase genes	%
P1/ cr#3	4,771	884	18.5
P1/ cr#6	1,286	334	26.0
P2/ cr#3	7,738	1,670	21.6
P2/ cr#6	2,996	880	29.4

Supplementary Table 2. Distribution of TAC as a PAM in protospacers from newly acquired spacers. All spacers with more than 10 reads were analysed in regard to the PAM sequence in the respective protospacer. Column "locus and crRNA": locus into which the new spacer was integrated and crRNA, which was used for self-targeting (cr#3 and cr#6: crtl#3 or crtl#6 was used, respectively); column "number of reads": number of reads for all spacers; column "protospacer with TAC PAM": number of spacers that are derived from a protospacer with the PAM TAC; column "% TAC PAM": percentage of protospacers with TAC PAM.

**locus and crRNA**	**number of reads**	**protospacers withTAC PAM**	**% TAC PAM**
**chromosome**			
P1 cr#3	2,151	1,383	64
P1 cr#6	342	342	100
P2 cr#3	3,844	2,269	59
P2 cr#6	962	826	86
sum	7,299	4,820	66
**pHV4**			
P1 cr#3	607	607	100
P1 cr#6	166	166	100
P2 cr#3	1,180	1,146	97
P2 cr#6	423	423	100
sum	2,376	2,342	98,6
**pHV3**			
P1 cr#3	11	11	100
P1 cr#6	0	0	-
P2 cr#3	124	124	100
P2 cr#6	36	36	100
sum	171	171	100
**pHV1**			
P1 cr#3	121	121	100
P1 cr#6	0	0	--
P2 cr#3	260	260	100
P2 cr#6	63	63	100
sum	444	444	100
total sum	10,290	7,777	76

Supplementary Table 3. Hotspots from naïve adaptation. Hotspots seen for naïve adaptation were compared to hotspots found with targeting induced adaptation (upon low crRNA concentrations see also Figure 4 and Suppl. Figure 5). Some hotspots are similar whereas some are not. Column "hotspot naïve": hotspot numbers as shown in Supp. Figure 5; column "self-targeting": this hotspot is also found at low self-targeting conditions: yes or no; column "gene": gene(s) found at hotspot.

hotspot naïve	self-targeting	gene
1	yes	HVO_0276, ISH5-type transposase ISHvo11
2	no	HVO_0638, conserved hypothetical protein
3	no	HVO_0965, thymidine phosphorylase
4	yes	HVO_1429, hypothetical protein, homolog to pHK2-ORF6
5	yes	rRNA genes (HVO_1728-HVO_1732)
6	no	HVO_2081, pectin lyase domain protein
7	yes	vicinity of *orc11* and *orc14* genes (HVO_2289, 2293-2302)
8	no	HVO_2339 sensor box histidine kinase
9	yes	HVO_2397-2400, ABC-type transport system periplasmic substrate-binding protein (probable substrate zinc), small CPxCG-related zinc finger protein
10	no	HVO_2669-2683, flavodoxin domain protein, FAD-dependent oxidoreductase (GlcD/DLD_GlcF/GlpC domain fusion protein), aminotransferase class V (serine--pyruvate aminotransferase / alanine--glyoxylate aminotransferase), NRAMP family transport protein MntH (probable substrate manganese), histidinol dehydrogenase, HesB/IscA family iron-sulfur cluster assembly accessory protein, HTH domain protein, dodecin, cons. hypothetical proteins

Supplementary Table 4. Number of spacers acquired by naïve adaptation in H119 and Δ*cas6b*. See also Figure 11. Column "strain": strain from which genomic DNA was isolated for analysis; column "plasmid": plasmid present in strain; column "CRISPR locus": CRISPR locus, in which spacers were identified; column "Number of spacers acquired from the plasmid": number of spacer acquired from plasmid pTA927.

Strain	Plasmid	CRISPR locus	Number of total Spacers	Number of spacers acquired from the plasmid pTA927
H119	pTA927	P1	0	0
		P2	47	0
	pTA927-cas412	P1	273	21
		P2	16	0
Δ*cas6b*	pTA927	P1	0	0
		P2	84	0
	pTA927-cas412	P1	82	18
		P2	243	22

Supplementary Table 5. *E. coli* and *H. volcanii* strains used in this study.
The name, genotype and source of each strain is listed.

**Strains**	**Genotype**	**Source/ Reference**
DH5α	F- φ80*lac*ZΔM15 Δ(*lac*ZYA-*arg*F) U169 *rec*A1 *end*A1 *hsd*R17 (rk-, mk+) *gal*- *pho*A *sup*E44 λ- *thi*-1 *gyr*A96 *rel*A1	(invitrogen)
H119	ΔpHV2,Δ*pyrE2*, Δ*leuB*, Δ*trpA*	(1)
HV32	ΔpHV2,Δ*pyrE2*, Δ*leuB*, Δ*trpA*, ΔHVO_2,385,045–2,386,660, ΔHVO_pHV4: 204,834-218,566	(2)
HV35	ΔpHV2, Δ*pyrE2*, Δ*trpA*, Δ*leuB*, ΔHVO_2.385.045–2.386.660::p.tnaA,*cas6b*, *cas8b*, *cas7*,*cas5*, t.syn; ΔHVO_pHV4: 204.834-218.566	Vogel et al., submitted
Δ*cas6* (HV50)	ΔpHV2,Δ*pyrE2*, Δ*leuB*, Δ*trpA*, Δ*cas6b*	(3)
WR536	ΔpHV2,Δ*pyrE2*, Δ*trpA*	(4)

Supplementary Table 6. Plasmids used in this study.

Plasmid	Relevant properties	Source/Reference
pTA231	shuttle vector with *trpA* marker and pHV2 replication origin	(5)
pTA232	shuttle vector with *leu*B marker and pHV2 replication origin	(5)
pMA-RQ-telecrRNA19	*E. coli plasmid* containing the promoter, crRNA without 3' handle flanked by t-elements, and terminator, expressing the crRNA against spacer C1	(6)
pMA-RQ-crtI#6	*E. coli* plasmid containing the promoter, crRNA without 3' handle flanked by t-elements, and terminator, expressing the crRNA against the coding strand of *crtI*	this study
pTA232-crtI#3	high copy plasmid containing the promoter, spacer sequence flanked by *Haloferax* repeats and terminator, expressing a crRNA against the template strand of *crtI*	(7)
pTA232-tele-crtI#3	high copy plasmid containing the promoter, the mature crRNA (5´ handle, spacer sequence and 3´ trailer) flanked by t-elements, and terminator, expressing a crRNA against the template strand of *crtI*	(7)
pTA352-crtI#3	low copy plasmid containing the promoter, spacer sequence flanked by *Haloferax* repeats and terminator, expressing a crRNA against the template strand of *crtI*	(7)
pTA232-crtI#6	plasmid containing the promoter, spacer sequence flanked by *Haloferax* repeats and terminator, expressing a crRNA against the coding strand of *crtI*	this study
pTA927-p.tnaA-cas4-1-2	plasmid containing the *p.tnaA* promoter, the genes *cas4*, *cas1* and *cas2*, and a terminator	this study
pTA232-p.fdx-mrr	plasmid containing the *p.fdx* promoter, the *mrr* gene and a terminator	this study
pTA231-p.fdx-mrr	plasmid containing the *p.fdx* promoter, the *mrr* gene and a terminator	this study
pTA231-p.fdx-mrr-NFLAG	plasmid containing the *p.fdx* promoter, the *mrr* gene and a terminator, the Mrr endonuclease is expressed as a N-FLAG-fusion protein	this study
pRL3	plasmid containing the HEN recognition site with 250 bp flanking regions, cloned into pTA 354	(8)
pTA409-PAM9-CSp1	plasmid containing the sequence of the first spacer of CRISPR locus C with the PAM ACT	(9)

Supplementary Table 7. Oligonucleotides used in this study.

Name	Sequence
crtI#6iPCRup	CCGGCGCTACCCCTGCTCGCTTCAACTACCGATCAA
crtI#6iPCRdo	CCCAACCTCCTGTGCGGCACCGATATTGGTATGGCA
P1Leader-aussen-fw	GACGCACCACAACCGGCCGACTGA
P1.1-rev	GAGGTCGCCGGTCGAGATGCCTGC
P1.3-rev	TGGACCCCGGCTTCGTCGAGGAC
P2leader-innen-fw	CGTCTACCCCCGTGAATTCGGACGG
P2Leader-aussen-fw	GCGTCAACCGTGAGCGAGTCGGAC
P2.1-rev	CCCGGCAGAACGTCCACCCCGA
P2.3-rev	GTGTGATTCGATACGCGACACCACCGA
CLeader-aussen-rev	ACTGCCCGAGTGGTTCTCGACACCGCCAGTG
C.2-fw	CTTCGAAGTCGCCGCAGACGTGCGT
C.3-fw	CTTCGAAGTCGCCGCAGACGTGCGT
P1-fw	CCGTCTACCCCCGTGAATTCGGACGG
P1-fw-Ad	TCGTCGGCAGCGTCAGATGTGTATAAGAGACAGGTCTACCCCCGTG AATTCGGA
P1-fw-Ad-N	TCGTCGGCAGCGTCAGATGTGTATAAGAGACAGNGTCTACCCCCGT GAATTCGGA
P1-fw-Ad-NN	TCGTCGGCAGCGTCAGATGTGTATAAGAGACAGNNGTCTACCCCCG TGAATTCGGA
P1-fw-Ad-NNN	TCGTCGGCAGCGTCAGATGTGTATAAGAGACAGNNNGTCTACCCCC GTGAATTCGGA
P1-rev-Ad	GTCTCGTGGGCTCGGAGATGTGTATAAGAGACAGGTCGCCGGTCGA GATGCCT
P1-rev-Ad-N	GTCTCGTGGGCTCGGAGATGTGTATAAGAGACAGNGTCGCCGGTCG AGATGCCT
P1-rev-Ad-NN	GTCTCGTGGGCTCGGAGATGTGTATAAGAGACAGNNGTCGCCGGTC GAGATGCCT
P1-rev-Ad-NNN	GTCTCGTGGGCTCGGAGATGTGTATAAGAGACAGNNNGTCGCCGGT CGAGATGCCT
P2-fw-Ad	TCGTCGGCAGCGTCAGATGTGTATAAGAGACAGCGTCTACCCCCGT GAATTCG
P2-fw-Ad-N	TCGTCGGCAGCGTCAGATGTGTATAAGAGACAGNCGTCTACCCCCG TGAATTCG
P2-fw-Ad-NN	TCGTCGGCAGCGTCAGATGTGTATAAGAGACAGNNCGTCTACCCCC GTGAATTCG
P2-fw-Ad-NNN	TCGTCGGCAGCGTCAGATGTGTATAAGAGACAGNNNCGTCTACCCC CGTGAATTCG
P2Neu-rev	GTCTCGTGGGCTCGGAGATGTGTATAAGAGACAGGAGGATTGGGGA GATAATTCCG
P2Neu-N-rev	GTCTCGTGGGCTCGGAGATGTGTATAAGAGACAGNGAGGATTGGGG AGATAATTCCG
P2Neu-N-rev	GTCTCGTGGGCTCGGAGATGTGTATAAGAGACAGNNGAGGATTGGG GAGATAATTCCG
P2Neu-N-rev	GTCTCGTGGGCTCGGAGATGTGTATAAGAGACAGNNNGAGGATTGG GGAGATAATTCCG
Hindcas4Start	TATATAAAGCTTATGAGTTCAACTGACGTCGTC

cas2StoppBamHI	TATATAGGATCCTCAGGTAAACCGACTCCCCGGTTCG
P1Leader-fw-HindIII	TATATAAAGCTTAGATTCGTTTCCGTCGACCCTCGGGGGTAC
CLeader-fw-HindIII	TATATAAAGCTT CCCGACGTTTTCGTCGACCCCCCGGG
LocusShort-rev-BamHI	TATATAGGATCCAAGGGTTCGTCTGAAACCATCGCCTTGAGCGCGAT
deletion1leaderrev	CCTCTCCTCACGACCAGCGGTGC
spacer crRNA crtl#3	CTCTGCGACCAGGTCGTCTCCGACGCCGACTACGCC
spacer crRNA crtl#6	CGGCGTGTCCTCCAACCCGGCCGCGATGGGGACGAG
3'-mrr-XbaI	TATATATCTAGATTAACTCCCGTTCCAAACGTATCGTTTTT
5'-mrr-NdeI	TATATACATATGATGGACGCTGCCGCCGCGACC
5-mrr-SnaBI	TATATATACGTAATGGACGCTGCCGCCGCGACC
3-mrr-XbaI	TATATATCTAGATTAACTCCCGTTCCAAACGTATCGTTTTT
5-mrr-NdeI	TATATACATATGATGGACGCTGCCGCCGCGACC
IS124	GATGTGCTGCAAGGCGATTA
IS125	AGTGAGCGCAACGCAATTA
270	GGGTCGACGGAAACGTTGAT
271	AATTGGACCCCGGCTTCG
270	GGGTCGACGGAAACGTTGAT
272	TGTGATTCGATACGCGACAC

References

1. Allers, T., Ngo, H.-P., Mevarech, M., and Lloyd, R. G. (2004) Development of additional selectable markers for the halophilic archaeon Haloferax volcanii based on the leuB and trpA genes. *Applied and environmental microbiology* **70**, 943–953
2. Stachler, A. E., Schwarz, T. S., Schreiber, S., and Marchfelder, A. (2019) CRISPRi as an efficient tool for gene repression in archaea. *Methods*
3. Brendel, J., Stoll, B., Lange, S. J., Sharma, K., Lenz, C., Stachler, A.-E., Maier, L.-K., Richter, H., Nickel, L., Schmitz, R. A., Randau, L., Allers, T., Urlaub, H., Backofen, R., and Marchfelder, A. (2014) A complex of Cas proteins 5, 6, and 7 is required for the biogenesis and stability of clustered regularly interspaced short palindromic repeats (crispr)-derived rnas (crrnas) in Haloferax volcanii. *The Journal of biological chemistry* **289**, 7164–7177
4. Abu-Qarn, M., Eichler, J., and Sharon, N. (2008) Not just for Eukarya anymore: protein glycosylation in Bacteria and Archaea. *Curr Opin Struct Biol* **18**, 544-550
5. Allers, T., and Mevarech, M. (2005) Archaeal genetics - the third way. *Nature reviews. Genetics* **6**, 58–73
6. Maier, L.-K., Stachler, A.-E., Saunders, S. J., Backofen, R., and Marchfelder, A. (2015) An active immune defense with a minimal CRISPR (clustered regularly interspaced short palindromic repeats) RNA and without the Cas6 protein. *The Journal of biological chemistry* **290**, 4192–4201

7. Stachler, A.-E., Turgeman-Grott, I., Shtifman-Segal, E., Allers, T., Marchfelder, A., and Gophna, U. (2017) High tolerance to self-targeting of the genome by the endogenous CRISPR-Cas system in an archaeon. *Nucleic Acids Research* **45**, 5208-5216
8. Naor, A., Lazary, R., Barzel, A., Papke, R. T., and Gophna, U. (2011) In vivo characterization of the homing endonuclease within the polB gene in the halophilic archaeon Haloferax volcanii. *PLoS One* **6**, e15833
9. Fischer, S., Maier, L.-K., Stoll, B., Brendel, J., Fischer, E., Pfeiffer, F., Dyall-Smith, M., and Marchfelder, A. (2012) An archaeal immune system can detect multiple protospacer adjacent motifs (PAMs) to target invader DNA. *The Journal of biological chemistry* **287**, 33351–33363
